# Extensive gain and loss of photosystem I subunits in chromerid algae, photosynthetic relatives of apicomplexans

**DOI:** 10.1038/s41598-017-13575-x

**Published:** 2017-10-16

**Authors:** Roman Sobotka, Heather J. Esson, Peter Koník, Eliška Trsková, Lenka Moravcová, Aleš Horák, Petra Dufková, Miroslav Oborník

**Affiliations:** 1 Centre Algatech, Institute of Microbiology, Czech Academy of Sciences, Třeboň, Czech Republic; 2 Institute of Parasitology, Biological Centre, Czech Academy of Sciences, České Budějovice, Czech Republic; 3 Faculty of Science, University of South Bohemia, České Budějovice, Czech Republic

## Abstract

In oxygenic photosynthesis the initial photochemical processes are carried out by photosystem I (PSI) and II (PSII). Although subunit composition varies between cyanobacterial and plastid photosystems, the core structures of PSI and PSII are conserved throughout photosynthetic eukaryotes. So far, the photosynthetic complexes have been characterised in only a small number of organisms. We performed *in silico* and biochemical studies to explore the organization and evolution of the photosynthetic apparatus in the chromerids *Chromera velia* and *Vitrella brassicaformis*, autotrophic relatives of apicomplexans. We catalogued the presence and location of genes coding for conserved subunits of the photosystems as well as cytochrome b_6_f and ATP synthase in chromerids and other phototrophs and performed a phylogenetic analysis. We then characterised the photosynthetic complexes of *Chromera* and *Vitrella* using 2D gels combined with mass-spectrometry and further analysed the purified *Chromera* PSI. Our data suggest that the photosynthetic apparatus of chromerids underwent unique structural changes. Both photosystems (as well as cytochrome b_6_f and ATP synthase) lost several canonical subunits, while PSI gained one superoxide dismutase (*Vitrella*) or two superoxide dismutases and several unknown proteins (*Chromera*) as new regular subunits. We discuss these results in light of the extraordinarily efficient photosynthetic processes described in *Chromera*.

## Introduction

Photosystem I (PSI) and photosystem II (PSII) are large pigment-protein complexes, assembled from tens of subunits and harboring various cofactors, embedded in the thylakoid membranes of oxygenic phototrophs (cyanobacteria, algae and plants). Using energy from photons, PSII generates redox potential that is high enough to oxidise water, a virtually unlimited source of electrons in nature. Electrons withdrawn from water are passed via plastoquinone molecules to cytochrome *b*_6_ *f*, where their energy is used to pump protons into the thylakoid lumen. The low-energy electrons are then transferred to the lumenal protein plastocyanin. At this point PSI must provide very strong redox potential in order to transfer electrons from plastocyanin to stromal ferredoxin. The redox potential of this electron carrier is sufficient to reduce NADP to NADPH in the stroma and thus provide reducing power for the Calvin-Benson cycle. The proton gradient generated by the photosynthetic electron transport chain powers ATP synthase, an enzyme that converts ADP to ATP.

Over 3.5 billion years photosystems probably evolved from a simple homodimeric structure into complicated and very efficient nano-machines^1^. All modern oxygenic photosynthetic organisms, however, share the common core structures of PSI and PSII, whose overall configurations are ancient and apparently of cyanobacterial origin. The PSI core is assembled from two large and closely related proteins, PsaA and PsaB, which form a heterodimer. Together PsaA and PsaB bind about 100 chlorophyll molecules and other cofactors (e.g. phylloquinone and a Fe-S cluster) required for electron transfer. The small stromal subunits PsaC, PsaD and PsaE form the binding site for ferredoxin and PsaC binds another Fe-S cluster serving as a terminal electron acceptor. About 15 small membrane subunits are attached to PsaA/B, most of which are highly conserved and present in the PSI complexes of all lineages of oxygenic phototrophs (PsaF, PsaH, PsaK, PsaI and PsbL). The core of the PSII complex consists of four large proteins - D1, D2, CP47 and CP43 - that bind most of the chlorophyll molecules (~30) and most of the other cofactors. These large subunits are joined by a number of small, single helix proteins (for example, PsbI, PsbH, PsbT, and PsbZ); two small proteins (PsbE and PsbF) together bind a heme molecule. At the lumenal site of PSII several hydrophilic proteins are assembled around a manganese cluster, where water molecules are split into oxygen and protons.

PSI composition in different taxa varies mostly due to the chlorophyll-binding, light-harvesting complexes associated with PSI that are present in eukaryotes but absent in cyanobacteria, where PSI is associated with phycobilisomes. The number and arrangement of antenna proteins and their pigment compositions vary between plastid lineages (e.g. refs^2,3^). There are also differences in numbers of small subunits; red algal-derived plastids, for example, lack PsaG and PsaH subunits^4^. The evolution of eukaryotic (plastid) PSI also eliminated the oligomeric (trimeric) form of PSI common in cyanobacteria; plastid PSIs are typically monomeric^5^. Similar to PSI, the eukaryotic PSII became surrounded by light-harvesting complexes and the number and composition of extrinsic lumenal proteins differs in cyanobacteria, algae and plants. However, the typical dimeric structure of plastid PSII remains highly conserved and closely resembles cyanobacterial PSII^6–8^.

The number of taxa where the differences in photosystem protein composition have been examined and compared is relatively small – studies have been confined mostly to higher plants, *Chlamydomonas*, and cyanobacteria, although research has recently extended to red algae and stramenopiles^4,9–13^. Considering the tremendous diversity of photosynthetic organisms and the complexity of plastid evolution in eukaryotes, our current understanding of photosystem composition is extremely limited.

Extant plastid diversity is the product of a complicated history involving multiple endosymbiotic events. A single-celled eukaryote engulfed and retained a cyanobacterial prey cell giving rise to glaucophytes, red algae, green algae and plants (primary endosymbiosis). In two distinct secondary endosymbiotic events, green algal prey cells were engulfed by the ancestor of the chlorarachniophytes and by a phago-heterotrophic euglenid, respectively. Secondary endosymbiosis involving a red algal prey cell has occurred at least once in evolutionary history, giving rise to the plastids in stramenopiles, dinoflagellates possessing peridinin, haptophytes, cryptophytes, and apicomplexans^14–18^; however, hypotheses involving independent secondary^19^ or tertiary endosymbioses are becoming more plausible (e.g. ref.^20^). Endosymbiosis invariably led to the reduction of endosymbiont (plastid) genomes as genes were gradually incorporated into the host nucleus^21–23^. In non-photosynthetic plastid-containing lineages the loss of photosynthesis has been accompanied by physical reduction of the plastid. An important example is that of the apicomplexans, unicellular parasites that are the causative agents of malaria, toxoplasmosis, and other human and livestock diseases. Most apicomplexans, most notably *Plasmodium* and *Toxoplasma*, host a relict plastid (the “apicoplast”) in their cells^24–26^. Their closest, extant, photosynthetic relatives are *Chromera velia* and *Vitrella brassicaformis* (hereafter *Chromera* and *Vitrella*), unicellular algae that are associated with stony corals^27–30^. The fully sequenced plastid genome of *Chromera* revealed some evolutionary oddities, such as two essential thylakoid proteins (PsaA and AtpB) each split into two smaller proteins, and an apparently linear genome^28,31^. The split PsaA subunit does not impede the function of PSI, which exhibits extremely high photochemical trapping efficiency^32^.

Transmission electron micrographs of *Chromera* PSI reveal a typical structure which is bound to light-harvesting complexes closely related to the PSI-antennae supercomplexes of red algae^33,34^. *Chromera* also possesses a clade of unique antennae proteins, termed “Chromera Light Harvesting” complexes that exhibit similarities to harvesting complexes from xanthophytes^33,34^. This is consistent with previous research indicating an ochrophyte origin of chromerid plastids^35^. The photosynthetic pigment compliment of *Vitrella* - chlorophyll-*a* (but not chlorophyll-*c*), violaxanthin, vaucheriaxanthin, β-carotene, and an isofucoxanthin-like pigment – closely resembles that of the eustigmatophyte *Nannochloropsis limetica* ^27,29^. The pigment complement in *Chromera* is similar to that in *Vitrella*, but *Chromera* lacks vaucheriaxanthin and possesses isofucoxanthin instead^27^.

Photosynthesis studies have revealed *Chromera*’s high capacity for adaptation to different light conditions, allowing it to maintain high rates of photosynthesis and growth without succumbing to photoinhibition^36–38^. In order to expand our understanding of the relationship between photosystem structure and function in chromerid algae, we used biochemical methods to identify the components of PSI. To put our results in an evolutionary context, we analysed the sequences of 55 proteins integral to the structure and function of the four thylakoid membrane complexes from *Chromera*, *Vitrella*, stramenopiles, red algae, green algae, plants, glaucophytes and cyanobacteria.

## Results and Discussion

### Loss of plastid proteins associated with photosynthetic complexes

**Figure 1:**
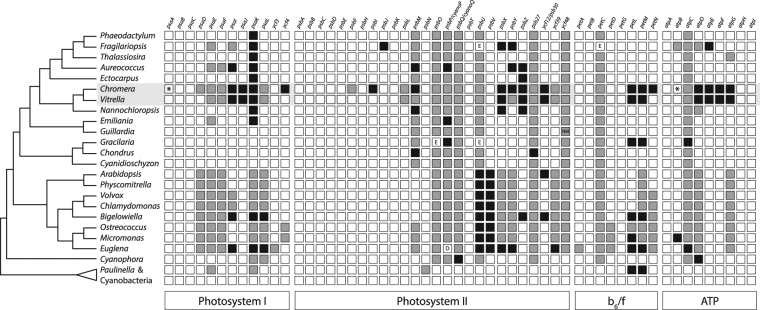
Nuclear transfer and loss of genes coding for thylakoid membrane complex proteins in cyanobacteria and eukaryotes. *Chromera* has experienced a greater degree of gene loss than other eukaryotic taxa. White squares denote plastid-encoded genes; gray squares, nuclear-encoded genes; black squares, absent genes. In *Chromera* both *psaA* and chloroplast *atpB* are split into two segments (indicated with an asterisk). E: protein sequence is derived from EST data. D: protein was directly sequenced. NM: protein is encoded in the nucleomorph.

To obtain a broader view on the evolution of photosynthetic complexes we retrieved the amino acid sequences of 55 proteins associated with PSI, PSII, cytochrome *b*_6_ *f* and ATP synthase from sequence databases (genes and accession numbers are given in Table S1, Supplementary Material). We focused specifically on subunits documented both in cyanobacteria and in eukaryotes. Sequences were obtained from the cyanobacteria *Prochlorococcus marinus*, *Synechococcus elongatus*, *Thermosynechococcus elongatus*, *Anabaena* spp., and *Microcystis* spp.; the chromerids *Chromera* and *Vitrella;* the rhizarians *Paulinella chromatophora* and *Bigelowiella natans*; the glaucophytes *Cyanophora paradoxa* and *Glaucocystis nostochinearum*; the red algae *Gracilaria* spp., *Chondrus crispus* and *Cyanidioschyzon merolae*; the green algae *Chlamydomonas reinhardtii*, *Volvox carteri*, *Micromonas* spp. and *Ostreococcus* spp.; the plants *Arabidopsis thaliana* and *Physcomitrella patens*; the stramenopiles *Aureococcus anophagefferens*, *Ectocarpus siliculosus*, *Nannochloropsis gaditana*, *Phaeodactylum tricornutum*, *Thalassiosira pseudonana* and *Fragilariopsis cylindrica*; the haptophyte *Emiliania huxleyi*; the cryptophyte *Guillardia theta;* and the euglenophyte *Euglena gracilis*. Proteins were scored as either nuclear-encoded, plastid-encoded, or absent (Fig. 1). There were several observable phylogenetic patterns of transfer and loss in certain genes and taxa, particularly when *Paulinella* is excluded from consideration. *Chromera*, however, appears to have lost an unusually high number of genes: from PSI, *psaI*, *psaJ*, *psaK*, and *ycf4*; from PSII, *psbI*, *psbM*, *psbX*, *psbY*, *psbZ*, and *psb30*; from cytochrome b_6_/f, *petL*, *petM*, and *petN*; and from ATP synthase, *atpD*, *atpE*, *atpF*, and *atpG*.

The PsaI protein stabilises PsaL in the cyanobacterium *Synechocystis* ^39^; PsaL and PsaH form a docking site for light-harvesting complex II (LHCII) during state transitions in tobacco^40^ and the stability of this site relies on PsaI^41^. PsaI is present in all taxa considered here except for *Chromera*, *Vitrella*, *Aureococcus*, and the secondary green algae *Bigelowiella natans* and *Euglena gracilis* (Fig. 1). While *psaH* is absent from the red lineage (and from cyanobacteria, and thus not considered here^4^), *psaL* is present (although it has undergone transfer to the nucleus in *Chromera* and *Vitrella*; Fig. 1). PsaK, responsible for binding LHCI^42^, is absent in all secondary red algae (Fig. 1). These absences could be explained as a result of the different light harvesting requirements and associated pigment profiles of different plastid lineages^37,43^; they could, however, be an artefact resulting from our failure to detect divergent protein sequences. PsaJ appears to stabilise PsaF, which in turn facilitates electron transfer from plastocyanin to the PSI reaction center in *Chlamydomonas* and *Arabidopsis* ^44,45^. Interestingly, although *psaJ* is absent in *Chromera* and *Vitrella*, *psaF* is present in the nucleus of both (Fig. 1). PsaF, however, is predicted to influence the binding capabilities of PsaN^46^, a protein responsible for plastocyanin binding in plants^45^ but absent in the red lineage. It seems likely, therefore, that the interactions between PSI subunits differ in red plastids and the more widely studied green lineage. Ycf4 is involved in the biogenesis of PSI in green algae and plants^47,48^ and is absent only in *Chromera*. While we cannot rule out the possibility that *Chromera* Ycf4 is too divergent to detect using BLAST or PSI-BLAST, the protein is not essential in tobacco^49^ and it is therefore conceivable that Ycf4 is not required for PSI biosynthesis in *Chromera*.

PsbI and PsbM are small subunits of PSII thought to be associated with stability^50,51^ and dimerization of the photosystem^52^. PsbX, PsbY, PsbZ and Psb30 are peripheral PSII subunits that were also linked to photosystem stability and photoprotection^53–56^. The loss of *psbM* in *Chromera* is interesting in light of its role in dimerization (see below); this loss is shared with *Aureococcus*, *Nannochloropsis* and *Chondrus* (Fig. 1). While the nature of PSII oligomerization in *Aureococcus* and *Chondrus* is unknown, monomeric PSII has been observed in *Nannochloropsis oceanica* ^57^. Since the absence of the peripheral proteins in *Chromera* does not prevent efficient and adaptable photosynthesis^36–38^, *Chromera* must possess novel mechanisms, and possibly novel PSII subunits, to enable this efficiency. While no other taxon considered here lacks all of the four peripheral proteins missing in *Chromera*, eight other taxa – *Fragilariopsis*, *Aureococcus*, *Vitrella*, *Nannochloropsis*, *Arabidopsis*, *Bigelowiella*, and *Euglena* - lack one or more of them, suggesting that the protein infrastructure required for maintaining photosystem stability and light tolerance is evolutionarily flexible. Nevertheless, *Chromera* exhibits the highest degree of PSII gene loss: six gene losses in total.

PetL, PetM and PetN are small subunits of the cytochrome b_6_f complex. PetL appears to be involved in maintaining stability of the complex and functional conformation of the Rieske protein (PetC). While it has been shown to be non-essential for survival or photosynthesis in tobacco, the green alga *Chlamydomonas*, and the cyanobacterium *Synechocystis*, its loss can reduce b_6_f stability and the efficiency of electron transport^58–60^. PetM is a peripheral protein^61^ whose suppression prevents the accumulation of the b_6_f complex in young tobacco leaves^62^; in *Synechocystis*, on the other hand, interruption of the *petM* gene has no observable effect on b_6_f but does decrease PSI and phycobilisome content^63^. PetN is required for the stability of the b_6_f complex in tobacco and knockout mutants are incapable of carrying out photosynthesis^60,64^. The absence of *petL* and *petM* in *Chromera*, *Vitrella*, *Bigelowiella*, *Euglena* and *Paulinella* (Fig. 1), combined with the relatively minor effects of their suppression as described above, could indicate the presence of redundant, as yet undescribed stabilizing proteins in these taxa. *Chromera*, however, is the only taxon lacking *petN*, implying either a highly divergent sequence (entirely possible given PetN’s small size^64^), or a novel mechanism for maintaining b_6_f stability unique to *Chromera*.

The ATP synthase complex also exhibits extensive loss in chromerids (Fig. 1). Of particular interest are four genes encoding stabilizing and structural proteins in ATP synthase, absent in both *Chromera* and *Vitrella*: *atpD*, (also absent from the glaucophyte *Cyanophora paradoxa*), *atpE*, *atpF* (absent in *Nannochloropsis*), and *atpG*. The absence of these genes, present in almost all other eukaryotes surveyed here, should have serious and extensive consequences for the structure and function of ATP synthase – for example, in *Arabidopsis*, knockout of *atpD* resulted in seedling lethality^65^. The electron transport chain still functions in *Chromera* and *Vitrella*, however, and, in the case of *Chromera*, does so efficiently^36–38^. It seems extremely likely, therefore, that *Chromera* and *Vitrella* merely possess ATP synthase subunits with highly divergent amino acid sequences that have eluded previous similarity-based searches and annotation efforts^66^.

### Phylogenetic analysis of photosynthetic complex proteins

In order to provide a phylogenetic framework for interpreting our results, we constructed individual alignments for 55 proteins and concatenated these into a single alignment for phylogenomic analysis. Highly variable sites were excluded to form two datasets from this primary alignment: one site-rich (10 465 sites) and one site-poor (4872 sites). The alignments were analysed using both maximum likelihood (RAxML^67^) and Bayesian (PhyloBayes 3.3f^68^) methods. Although their resulting topologies differed, both analyses recovered the red lineage as a clade to the exclusion of cyanobacteria, glaucophytes, and the green lineage (Fig. S1, Supplementary Material). *Chromera* and *Vitrella* formed a well-supported clade within the stramenopiles in both analyses and were recovered together as a sister group to the eustigmatophyte *Nannochloropsis gaditana* in the site-rich analysis with moderate/high support (Fig. S1A). In the site-poor analysis *Nannochloropsis* was the sister taxon to the remaining stramenopiles and the chromerids, but this relationship was unsupported (Fig. S1B).

The branching of chromerids within the stramenopiles – and specifically with *Nannochloropsis* - is consistent with pigment composition^29^ and phylogenomic analyses of plastid genomes performed previously^35^. It also supports suggestions that a single endosymbiotic event involving a red algal prey cell is not the best explanation for extant red plastid diversity^20,69–72^. On the contrary, the distribution of secondary red plastids among eukaryotes is better explained as a result of multiple secondary endosymbiotic events involving a red algal endosymbiont^19,70^ or the lateral inheritance of a secondary red plastid via tertiary and/or quaternary endosymbiotic events in the relevant lineages^20^. If an ancestor of “chrompodellids” (i.e. the clade comprised of colpodellids and chromerids - neither group is itself monophyletic^23,73^) and apicomplexans obtained its plastid via higher order endosymbiosis involving a recent ancestor of *Nannochloropsis* (after its divergence from other stramenopiles), the topology of the true plastid tree would resemble our topology (which admittedly excludes the dinoflagellates) (Fig. S1). A common origin of the eustigmatophyte and chromerid/apicomplexan plastids is further supported by the similar pigment compositions of *Nannochloropsis limnetica* and *Vitrella*; namely, both possess chlorophyll *a* (and lack chlorophyll *c*, as does *Chromera*), violaxanthin, vaucheriaxanthin, and β-carotene^29^. It is important to note, however, that both *Chromera* and *Vitrella* are prone to long-branch attraction in phylogenetic analyses - this is so severe in plastid genomes that *Chromera* sequences have occasionally been omitted from analyses in order to minimise the risk of recovering inaccurate topologies due to long-branch attraction^35^. Moreover, the sequence of hypothesised evolutionary events required to produce the conserved SELMA translocation machinery, active in the outer two membranes of secondary red plastids, is arguably unlikely to have evolved independently in multiple eukaryotic lineages^74^. Nevertheless, ML phylogeny presented in this work is consistent with the late acquisition of a eustigmatophyte endosymbiont in the common ancestor of chromerids and apicomplexans.

Phylogenetic analysis inferred from proteins of photosystems suggests that the genes coding for subunits of photosynthetic machinery have been lost multiple times over evolutionary history, and are therefore not strictly necessary for a functioning photosynthetic electron transport chain. *Chromera* and *Vitrella*, in particular, have sustained several losses that have not occurred in the majority of other red plastids; these genes – namely *psaI*, *psaJ*, *ycf4*, *psbI*, *psbY*, *ycf12/psb30*, *petL*, *petM*, *petN*, *atpD*, *atpE*, *atpF* and *atpG* - are therefore likely to have been lost in a recent common ancestor of the chromerid plastids. This would constitute a relatively rapid loss of photosystem proteins when compared to gene loss patterns in other plastid lineages. It is intuitively tempting to explain the extensive gene losses in chromerids in terms of the evolution of parasitism and predation in their apicomplexan and colpodellid relatives, respectively. Indeed, the acquisition of a parasitic life cycle in the apicomplexan lineage is associated with gene loss rather than the development of evolutionary novelties^23,75^, so the reduction of the photosynthetic apparatus could have been the precursor to the multiple losses of photosynthesis that have occurred in the chrompodellid and apicomplexan lineage.

### Protein mass spectrometry of *Chromera* and *Vitrella* thylakoid membranes

Given the extensive loss of thylakoid complex proteins in both chromerids, we questioned whether the photosynthetic complexes in these algae are merely reduced or whether they contain novel, non-canonical subunits. Using 2D Clear-Native/SDS electrophoresis (CN/SDS-PAGE) combined with mass-spectrometry (MS) we demonstrated previously that the PSI subunit PsaA and ATP synthase subunit AtpB in *Chromera* are split into two smaller proteins^31^. Nevertheless, the identification of most of the protein spots was not possible because of the lack of genomic data. Annotations of the fully sequenced nuclear and chloroplast genomes, however, are now available^28,66^.

**Figure 2:**
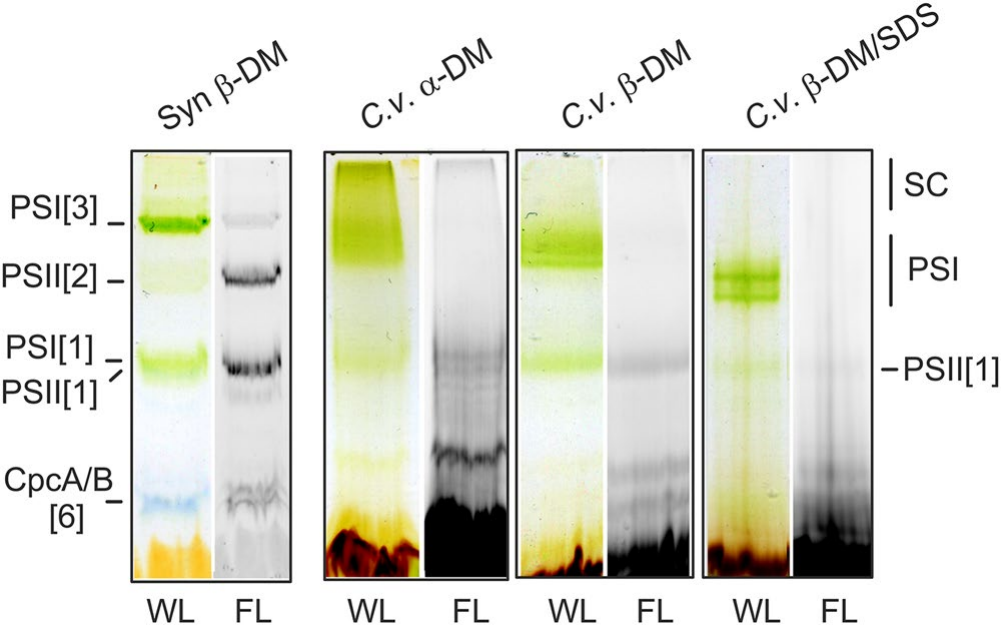
CN-PAGE of *Chromera* (*C.v*.) membrane complexes solubilised by α-DM, β-DM or β-DM with 0.4% SDS. For each line 15 μg of chlorophyll was loaded and the gel scanned under white light illumination (WL). Chlorophyll fluorescence emitted by PSII and by ‘free’ antenna proteins (FL) was detected by Fuji LAS 4000 after excitation by blue light. SC marks a PSI-PSII supercomplex observed only in the α-DM solubilised membranes (see Fig. 3); PSI marks a monomeric PSI supercomplex with (fucoxanthin-chlorophyll) light-harvesting antennae. Membranes (3 μg of chlorophyll) isolated from the cyanobacterium *Synechocystis* 6803 and solubilised with β-DM (*Syn* mem) were used to demonstrate the mobility of photosynthetic complexes: PSI[1] (~350 kDa) and PSI[3] (~1 MDa), monomer and trimer of PSI, respectively; PSII[2] (~700 kDa), dimer of PSII; CpcA/B[6], ~100-kDa heterohexamer of CpcA and CpcB phycobiliproteins.

**Figure 3:**
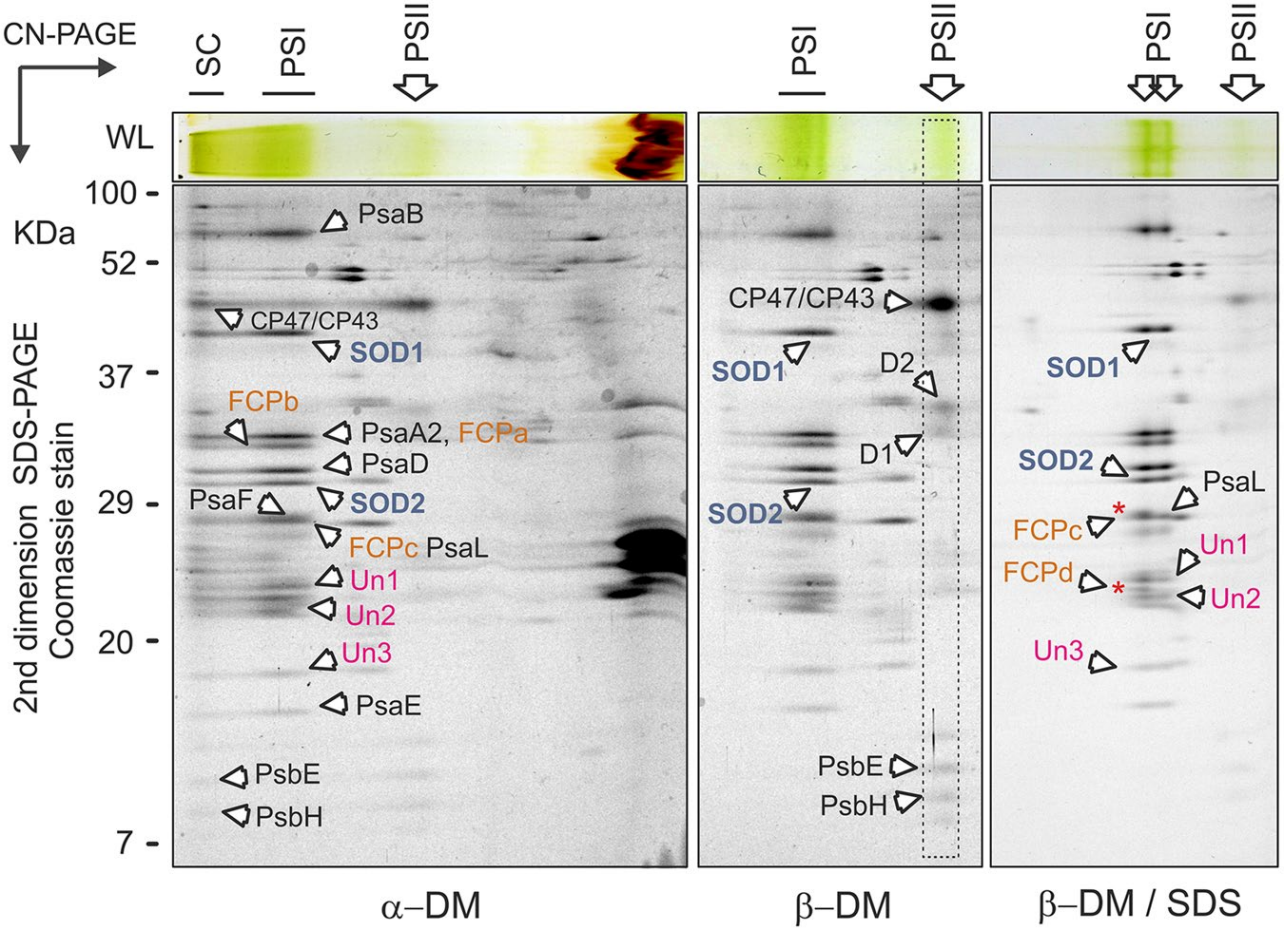
2D-PAGE of membrane complexes from *Chromera* solubilised by different detergents. Gel strips from CN-PAGE (see Fig. 2) were further separated in a second dimension by SDS-PAGE and stained with Coomassie blue; only the upper part of the gel is shown for the β-DM and β-DM/SDS samples. Individual spots were identified by MS. The PSI-PSII supercomplex (SC) observed after solubilisation with mild α-DM detergent was almost completely disrupted by β-DM solubilisation. Further addition of SDS resulted in the release of two antenna proteins from PSI (marked by red asterisks), however two superoxide dismutases (SOD1 and SOD2) and three other putative PSI subunits (Un1-3) remained bound to PSI core. All identified protein spots are listed in Table 1.

In order to characterise the organization of *Chromera* photosystems we used three different detergent systems to solubilise membrane complexes: a) n-dodecyl-α-maltoside (α-DM) that is recognised as a very mild detergent^76^, b) a conventional β-DM detergent that is generally less mild than α-DM^76^, and c) a relatively harsh mixture of β-DM and SDS to induce the partial disruption of protein complexes. After the separation of solubilised membrane complexes via CN-PAGE we obtained a different pattern for each detergent system (Fig. 2). Interestingly, a large assembly containing both PSI and PSII (see also Fig. 3) can be observed at the top of the CN gel after separation of the α-DM-solubilised complexes. It is notable that the chlorophyll fluorescence of PSII present in this putative supercomplex is strongly quenched, indicating that PSI acts as an energy trap for PSII fluorescence (Figs 2 and 3). The observed assembly is not preserved in β-DM and the individual PSI and PSII complexes are therefore better resolved. Adding SDS led to faster mobility of PSI due to the dissociation of some weakly bound subunits and the partial loss of PSII (Fig. 2).

**Figure 4:**
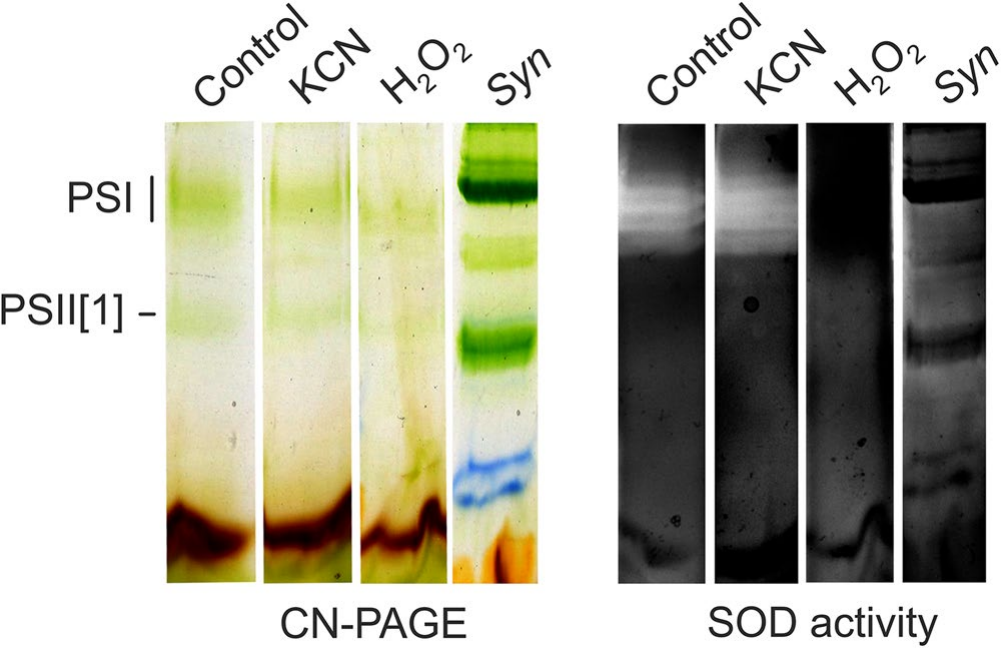
Detection of FeSOD activity in *Chromera* membrane complexes separated by CN-PAGE. Membrane were solubilised by β-DM and for each line 15 μg of chlorophyll was loaded. Membranes (3 μg of chlorophyll) isolated from the cyanobacterium *Synechocystis* 6803 were used as a negative control. After separation, the gel was stained for SOD activity essentially as described in ref.^96^. FeSOD enzymes are inhibited by peroxide but not by KCN, which however inhibits MnSOD enzymes. ZnSODs are inhibited neither by peroxide or KCN^96^.

Protein complexes were further separated in a second dimension by SDS-PAGE and individual putative subunits (co-migrating with PSI/PSII bands) were analysed by mass spectrometry (MS). As noted above, both PSI and PSII subunits can be found in a large structure at the top of the native gel with α-DM (Fig. 3), which suggests that in *Chromera* photosystems are tightly associated. Surprisingly, two different proteins showing high homology to Fe -superoxide dismutase enzymes (marked SOD1 and SOD2 in Fig. 3) were apparently co-migrating with PSI. Four SOD enzymes are annotated in the *Chromera* genome - two are MnSODs and two are FeSODs (SOD1/2). In order to confirm their putative identity, we queried the sequences of all four using SignalP^77^ and TargetP^78,79^ (see Supplementary Methods). The two FeSODs possess bipartite sequences consistent with plastid targeting (Supplementary Table S2). Sequences were also queried using HECTAR and HECTAR^SEC^80^. While only SOD2 (Cvel_3019) was identified as plastid-targeted by full HECTAR analysis, SOD1 (Cvel_7136) was predicted to possess both a signal peptide and a relatively high score for plastid targeting (Supplementary Table S3). Furthermore, HECTAR^SEC identified signal peptides in both sequences (Supplementary Table S4). A phylogenetic analysis of Fe- and MnSODs indicated that the *Chromera* FeSODs are not monophyletic; unfortunately, support values were too low to determine their evolutionary origin with certainty (Supplementary Fig. S3). To clarify whether SOD1/2 are true SOD enzymes, we measured SOD activity in a native gel after separation of *Chromera* membrane complexes. Indeed, we detected clear SOD activity co-localised with PSI. This activity was specifically inhibited by peroxide but not by KCN (Fig. 4). This implies that at least one of the two SOD proteins is an active FeSOD but, most likely, SOD1 and SOD2 work together as a heterodimer (see discussion).

In addition to SOD1 and SOD2, we identified three other putative PSI subunits, but none of these proteins (Un1-3, Fig. 3; Table 1) had any homologs revealed by BLASTing against the NCBI database. Their protein profiles on CryptoDB showed only signal peptides, transmembrane domains, and low complexity regions; Un1 also possessed prosite domain PS51257, a prokaryotic membrane lipoprotein lipid attachment site profile. Finally, four different fucoxanthin-chlorophyll antenna homologs (FCPa-d) appeared to be associated with PSI. After treatment with SDS two antenna proteins (FCPc, FCPd) were released from the PSI complex but otherwise this PSI appeared very stable and SOD1/2 proteins as well as Un1-3 proteins co-migrated with PSI even after solubilisation with SDS. In contrast to PSI we did not find any unexpected proteins associated consistently with PSII with comparable masses of those in *Chromera* or cyanobacterial monomeric PSIIs using CN-PAGE (~350 KDa; Fig. 2).

**Table 1:** List of *Chromera* proteins as identified by MS analysis of individual spots on 2D-PAGE of membrane complexes (Fig. 3) and the purified PSI (bold; Fig. 5).

Protein	Annotation^*a*^		Identified tryptic peptides/theoretical	
		Predicted mass (Da)	Membranes	**PSI**
**PsaB**	YP_003795270.2 (P)	102938	13/49	13/49
**AtpB2** ^*b*^	YP_003795258.2 (P)	61968		2/40
CP47	YP_003795264.2 (P)	61162	7/23	—
CP43	YP_003795274.1 (P)	53151	6/18	—
**PsaA2**	YP_003795268.2 (P)	49083	3/20	4/20
**SOD1**	Cvel_7136 (N)	41820	9/33	6/33
D2	YP_003795275.1 (P)	38569	6/14	—
D1	YP_003795256.1 (P)	38569	5/11	—
**FCPa**	Cvel_9928 (N)	31699	7/88	8/22
**FCPb**	Cvel_13255 (N)	32398	7/18	7/18
**PsaD**	Cvel_25206 (N)	30567	7/22	10/22
**SOD2**	Cvel_3019 (N)	29877	11/20	11/20
PsaF	Cvel_23764 (N)	35801	6/26	—
**PsaL**	Cvel_23765 (N)	29657	2/15	4/15
**FCPc**	Cvel_3431 (N)	26189	3/16	4/16
**PsaA1**	YP_003795268.2 (P)	32332	—	1/11
**Un1**	Cvel_30781 (N)	45879	3/37	5/37
FCPd	Cvel_1169 (N)	24034	6/18	—
**Un2**	Cvel_3217 (N)	22554	8/18	8/18
**Un3**	Cvel_6643 (N)	22517	2/18	5/18
**PsaE**	Cvel_5788 (N)	14402	4/11	4/11
PsbE	YP_003795269.1 (P)	9657	2/6	—
PsbH	YP_003795330.1 (P)	9348	3/3	—

*a* – CryptoDB database (http://cryptodb.org/cryptodb/) or NCBI database (https://www.ncbi.nlm.nih.gov/); N = nuclear encoded, P = plastid encoded.

*b* – probably a contaminating protein.

**Figure 5:**
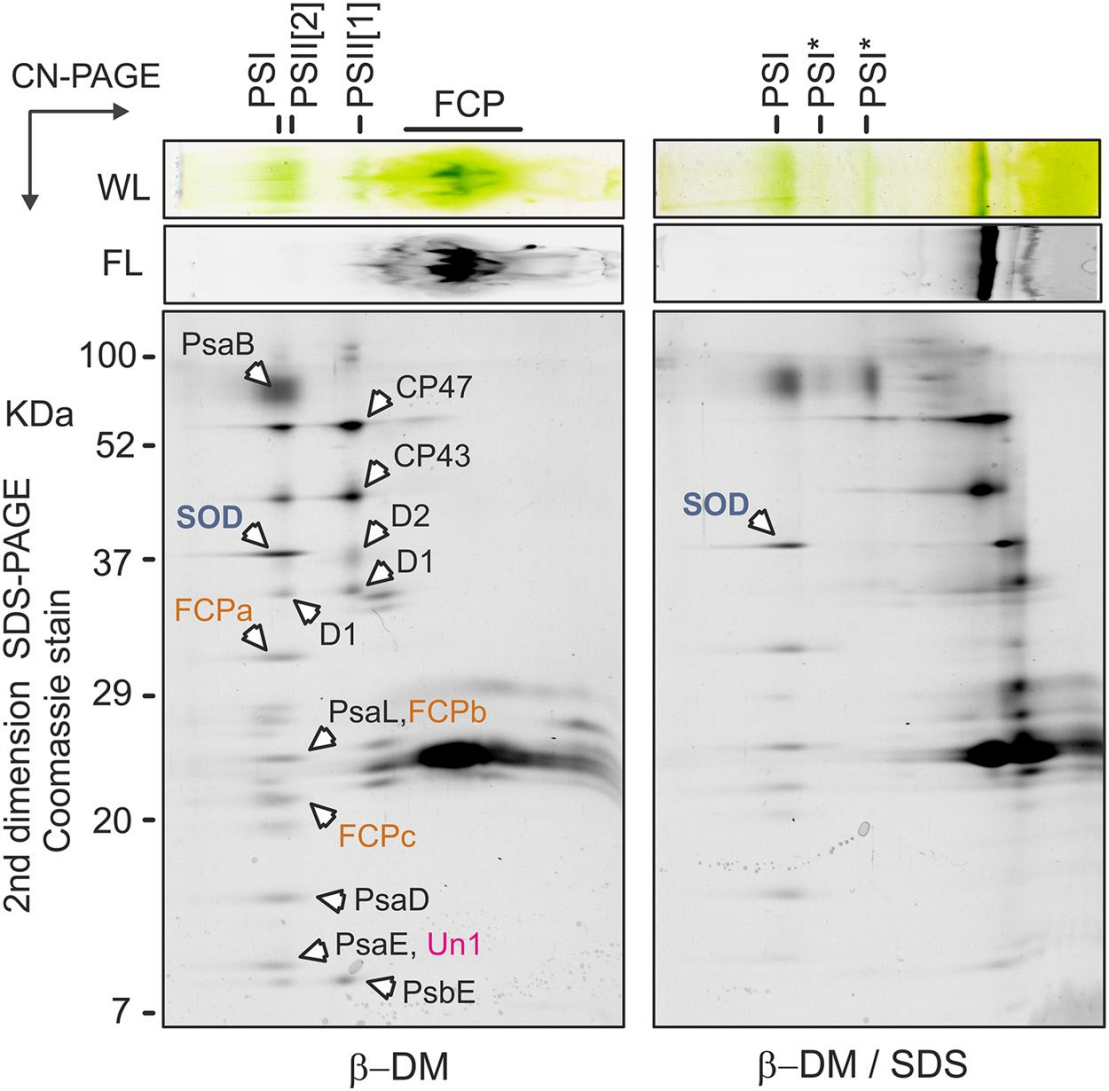
2D-PAGE of membrane complexes from *Vitrella* solubilised by β-DM and β-DM with 0.4% SDS. The gel was stained by Coomassie blue and individual spots were identified by MS (listed in Table 2). PSII[1] and PSII[2]; monomeric and dimeric PSII, respectively. PSI* marks partially disassembled PSI complexes.

Thylakoid complexes (including antenna proteins) isolated from *Vitrella* tended to smear on CN-gel (Fig. 5) and thus we failed to obtain CN-gel of α-DM solubilised membranes. However, MS analysis of β-DM and β-DM/SDS gels identified most spots belonging to PSI and PSII complexes unambiguously (Fig. 5; Table 2). In contrast to *Chromera*, the *Vitrella* PSII forms a dimer with a mass very similar to monomeric PSI – a pattern typical for plant photosystems^76^. The absence of a PSII dimer in *Chromera* is probably related to the loss of PsbM and PsbI subunits^52^; these proteins are coded in the *Vitrella* genome (Fig. 1). Similarly with *Chromera*, a FeSOD enzyme co-migrated with *Vitrella* PSI complex (Table 2) and this putative PSI-SOD interaction was preserved even after adding of SDS (Fig. 5). In addition, we detected an unknown protein co-migrating with *Vitrella* PSI (Table 2).

**Table 2:** List of proteins of *Vitrella* identified by MS on 2D CN/SDS PAGE of membrane complexes (see Fig. 4).

Protein	Annotation^*a*^	Predicted mass (Da)	Identified tryptic peptides/theoretical
**PsaB**	ADJ66611 (P)	85581	1/39
**SOD**	Vbra_10461 (N)	37353	5/28
**FCPa**	Vbra_17392 (N)	31914	3/16
**PsaL**	Vbra_18375 (N)	25913	1/11
**FCPb**	Vbra_9733 (N)	23552	2/16
**FCPc**	Vbra_1573 (N)	21039	6/11
**PsaD**	ADJ66635 (P)	13697	5/13
**PsaE**	Vbra_21597 (N)	13990	3/9
**Un1**	Vbra_13060 (N)	12117	2/4
**CP47**	ADJ66608 (P)	58872	12/30
**CP43**	ADJ66597 (P)	52667	4/31
**D2**	ADJ66652 (P)	41915	3/17
**D1**	ADJ66640 (P)	39086	4/15
**PsbE**	YP_003795269.1 (P)	12117	2/4

*a* - CryptoDB database (http://cryptodb.org/cryptodb/) or NCBI database (https://www.ncbi.nlm.nih.gov/).

### Subunit composition of the purified *Chromera* PSI

**Figure 6:**
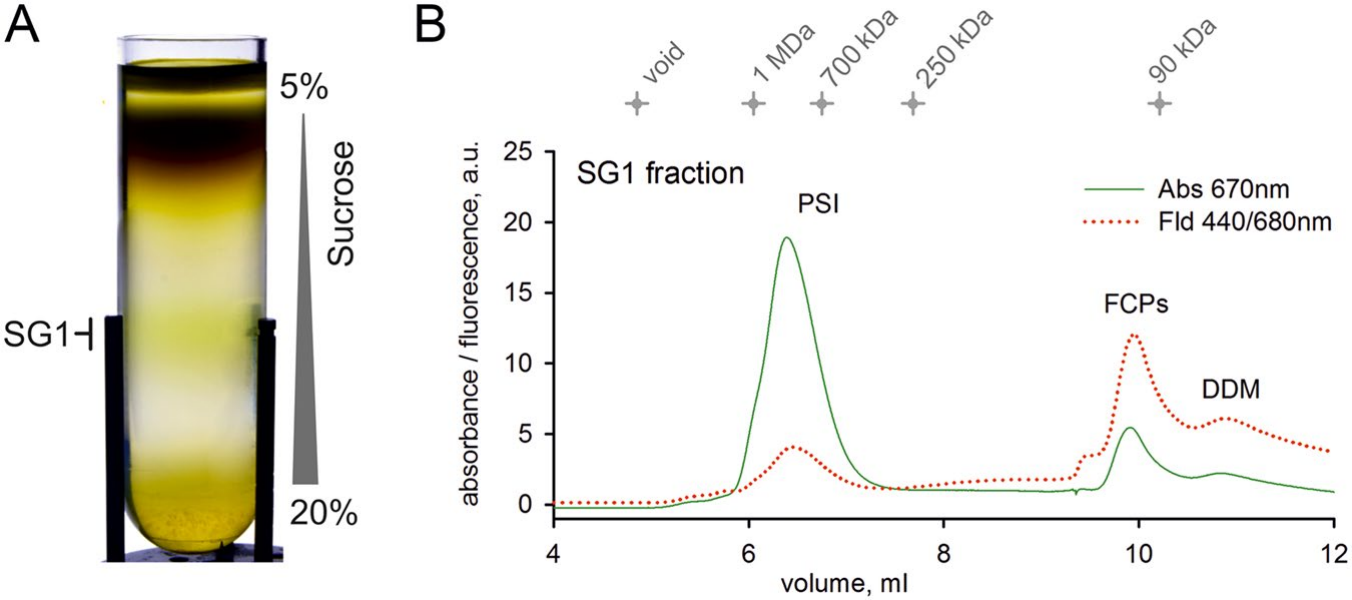
Isolation of the PSI complex from *Chromera*. (**A**) Solubilised membranes were loaded on a sucrose gradient and centrifuged at 75 000 x g for 18 hours; the SG1 (PSI) fraction was then collected and separated on a size-exclusion column (**B**). Eluted proteins and complexes were detected by a diode-array detector and a fluorescence detector set to 440/680 nm (excitation/emission wavelengths). Chlorophyll absorbance (670 nm) is shown by a green line and chlorophyll fluorescence is shown by a red dotted line; FCP, free antenna proteins. PSI complex, represented by peak 6-7.5 mL, was collected; see also Supplementary Fig. S3.

To confirm tight binding of FeSODs and other novel subunits to chromerid PSI we purified this complex from *Chromera* by a combination of sucrose gradient and size-exclusion chromatography (Fig. 6A,B). The isolated PSI had very low chlorophyll fluorescence (Fig. 6B) and exhibited an absorbance spectrum typical for algal (*Nannochloropsis*) PSI (Supplementary Fig. S3)^12^. The mass of *Chromera* PSI, as estimated from size-exclusion chromatography and CN-PAGE, was about 750 KDa (Fig. 6B, Supplementary Fig. S3). The migration of isolated PSI on CN-PAGE was similar to the PSI band in the β-DM-solubilised membrane complexes (compare Fig. 2 and Supplementary Fig. S3) implying that the purified complex is fairly intact.

**Figure 7:**
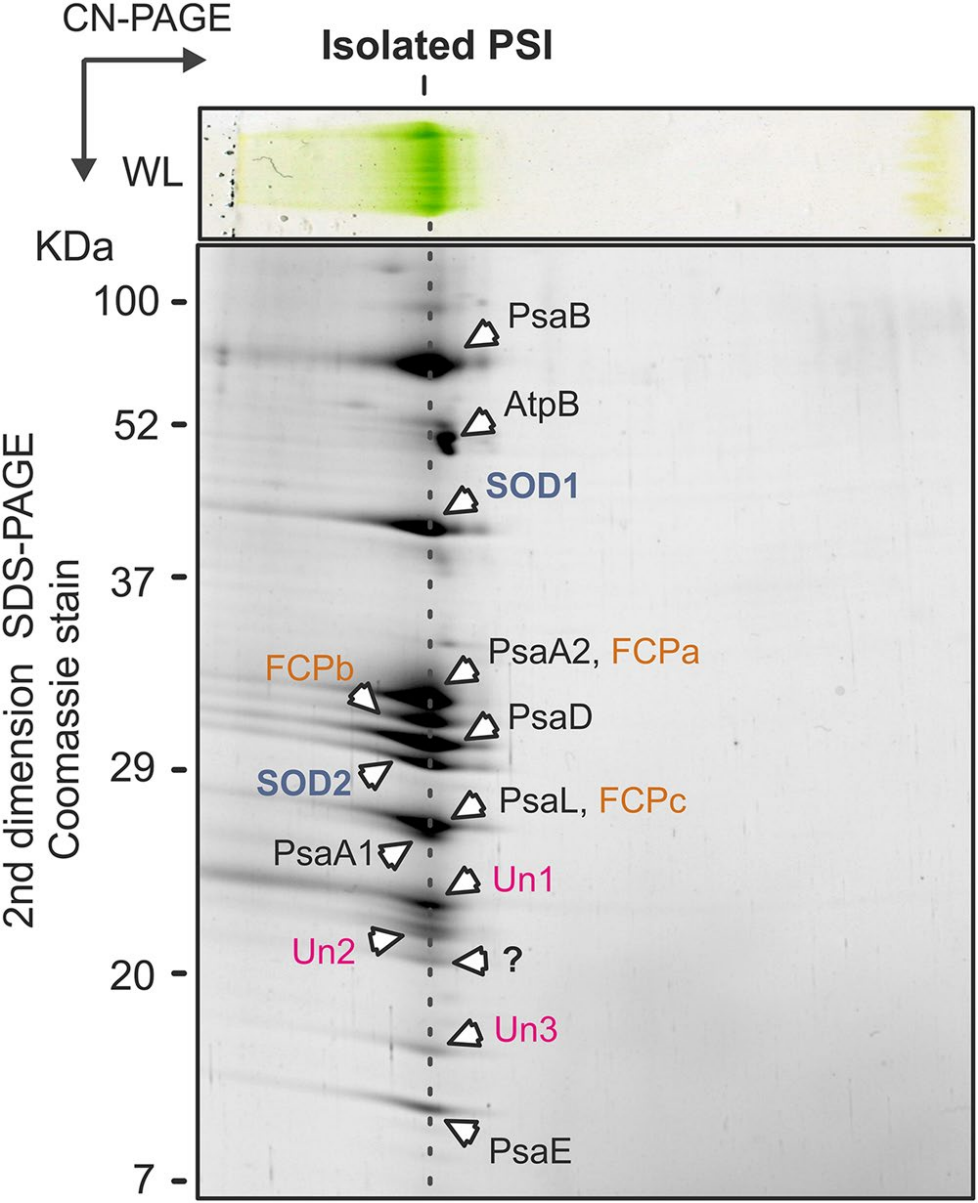
2D-PAGE of *Chromera* PSI complex purified by a combination of sucrose gradient and size-exclusion chromatography s described in Fig. 6. The gel was stained with Coomassie blue and individual spots were identified by MS (listed in Table 1).

The isolated PSI was separated on a 2D gel and all protein spots were cut and analysed by MS. Table 1 summarises identified PSI subunits. Although we did not detect PsaF and the FCPc antenna found previously in the separated membrane complexes (Fig. 3; Table 1) we confirmed that the SOD1/2 proteins and the three uncharacterised proteins Un1-3 are firmly attached to the PSI core. In addition, there was at least one more subunit with a mass of about 20 KDa which we failed to identify (Fig. 7). In the total membrane fraction and in the isolated PSI, all identified PSI subunits appear as abundant as the typical PSI subunits. We can therefore conclude that the *Chromera* PSI complex lost PsaI and PsaK, as indicated by our *in silico* analysis, but contains five new genuine subunits.

Although a new, small PSI protein (10 kDa) has been found in diatoms^10^, the addition of five new and relatively large subunits to PSI is unprecedented, particularly if none of these extra subunits seem to be related to antenna proteins^81^. In addition, after mild membrane solubilisation, the *Chromera* photosystems appear to be organised as a large assembly (Fig. 3). The existence of large molecular machinery in *Chromera* containing PSI and PSII together with b_6_f and ATP synthase would provide an attractive explanation for the observed structural changes in PSI including the PsaA split, the PSII monomerisation (lack of PsbM and PsbI), the loss of peripheral PSII subunits (PsbX/Y/Z/30) as well as for the loss of b_6_f subunits and the modification of ATP synthase (AtpB split, loss of AtpD/E/F/G). Indeed, it is a question how such a respirosome-like organisation would complicate the battery of regulatory mechanisms employed by oxygenic phototrophs to balance the excitation of photosystems and electron/proton fluxes^82^. It is known, however, that under high light conditions the majority of PSI complexes in *Arabidopsis thaliana* binds physically to PSII^83^ and supercomplexes containing PSI, cytochrome b_*6*_f and respiratory complex I are also well characterised in plants and green algae^84^. The formation of megadalton protein assemblies between photosystems and other thylakoid protein complexes therefore appears to be common in eukaryotic phototrophs. The packing of membrane complexes in *Chromera* might be much tighter due to structural changes in photosynthetic machinery and these lower dynamics are compensated for by the re-wiring of regulatory mechanisms.

SODs are crucial protective enzymes in oxidative phototrophs^85,86^. Together with catalases or peroxidases they detoxify superoxide anion radicals generated on the acceptor side of PSI. Although this reaction can be deleterious, it is generally utilised by autotrophs for the so-called water-water cycle as a sink for the excess electrons and protons produced by PSII. During this cycle O_2_^−^ generated by PSI is converted by SODs and peroxidases back to water while oxidising NADPH^82^. Under stress conditions in particular this cycle can account for a large electron flux^87^. Plant SODs are found in the vicinity of PSI^88^ and it is worth noting that two different plastid FeSOD enzymes form a heterodimer^89^. It is possible that such a heterodimer is a component of PSI in *Chromera*, whereas a FeSOD homodimer, as observed in the closely related *Plasmodium falciparum* cytosolic FeSODs^90^ (see Supplementary Fig. S2), is probably attached to *Vitrella* PSI (Fig. 5). Monomeric FeSODs are not found in nature^91^. The fact that *Chromera* FeSODs are transformed into regular PSI subunits indicates that the water-water cycle plays a prominent role in the regulation of photosynthesis. An interesting possibility is that chromerids can consume a significant amount of oxygen using the water-water cycle. Quigg and colleagues suggest that the very high carbon assimilation rates observed in *Chromera* are facilitated by the activation of the oxygen consumption process, which maintains high RuBISCO efficiency^36^. Organisation of the photosynthetic apparatus into a large supercomplex with extremely rapid electron transfer combined with a robust water-water cycle could provide a framework for the efficient and adaptable photosynthesis observed in *Chromera* ^37^. Although more biochemical and physiological work is needed to elaborate this hypothesis, these results, together with previous reports^31,92^ contribute to our growing understanding of the metabolic changes undergone in the ancestors of apicomplexans as they made the transition from alga to parasite.

## Materials and Methods

### Phylogenetic Analysis

NCBI and other databases (see Supplementary Table S1) were queried using blastp and tblastn from the ncbi+ package to retrieve genes coding for thylakoid membrane complex subunits (Supplementary Table S1). Blast results were parsed and respective homologues were extracted from our custom databases using perl scripts (available upon request). Datasets for each protein were aligned using MAFFT^93^. For each dataset, ambiguously aligned regions were manually deleted and the maximum likelihood (ML) phylogeny was inferred using RAxML^67^ with the gamma-corrected LG substitution matrix. The topology with the highest likelihood score was selected using the rapid bootstrapping algorithm (500 replicates). The branching support was assessed using thorough (“slow”) non-parametric bootstrap from 500 replicates using the model described above. Alternatively, Bayesian inference (BI) of single-gene phylogenies was performed using Phylobayes 3.3f^68^ using a combination of the empirical profile mixture model (C40) and the LG model. For each dataset, two chains were run until they converged (i.e. maximum observed discrepancy was 0.3 or lower) and the effective sample size of model parameters reached 100. Posterior probabilities computed after discarding the first 1/5^th^ of generations represent statistical support for branches.

A total of 55 protein datasets were then concatenated into a single dataset and analysed as described above with GTR for ML analysis and CAT + GTR for BI.

### Gene transfer and loss

Information for the location of each gene was gathered from its source database (see Supplementary Table S1). In the case of ambiguities, sequences were searched against an in-home BLAST database of relevant plastid genomes compiled from sequences publicly available on NCBI to ensure accurate annotation. Due to the divergent nature of chromerid plastids^28,35^ and extensive endosymbiotic gene transfer to the nucleus (Fig. 1), we have performed additional Position-Specific Iterated (PSI) BLAST screen against the complete set of predicted proteins available for *C*. *velia* and *V*. *brassicaformis* (http://cryptodb.org/) for all the chromerid genes missing from the standard blastp/tblastn searches (Fig. 1). Multiple sequence alignments of these genes (with the taxon sampling as defined by Fig. 1) were used as a ‘query’ in standalone PSI-BLAST (-in_msa option). We allowed the program to automatically estimate the number of iterations necessary for run convergence and set the significance threshold (e-value) to 1e-10.

### Strain cultivation

*Chromera* cells were grown in glass tubes in artificial seawater medium with supplementation of f/2 nutrients under continuous illumination with 100 μmol of photons s^−1^ m^−2^ (white light bulb) at 28 °C and bubbled with air. *Vitrella* cells were grown at 28 °C in a rotating 250-mL Erlenmeyer flask under irradiance of 20 μmol of photons s^−1^ m^−2^.

### Two dimensional electrophoresis and protein mass spectrometry

Cells were harvested and cellular membranes isolated as previously described^94^. Obtained membranes were solubilised with α-DM or β-DM for 1 h on ice (α/β-DM/chlorophyll = 20 w/w). Analysis of native membrane complexes was performed using CN electrophoresis as described by Wittig and colleagues^95^. Individual proteins in membrane complexes were resolved in the second dimension by SDS-PAGE in a 12–20% linear gradient polyacrylamide gel containing 7 M urea and protein identification by LC-MS/MS were carried out essentially as described previously^31^.

### Isolation of *Chromera* PSI

*Chromera* membranes prepared as described above were solubilised with β-DM for 1 h on ice (β-DM/chlorophyll = 20 w/w). After removing insoluble parts by centrifugation (65,000 × g, 20 min) solubilised protein complexes were separated by ultracentrifugation (141 000 × g for 18 h) on a 5–20% gradient of sucrose in thylakoid buffer containing 0.04% β-DM. A prominent green band with a migration expected for PSI was taken and concentrated 10-fold on a 100-kD cutoff microconcentrator (Millipore). The concentrated sample was immediately injected onto an Agilent-1200 HPLC machine and separated on a Yara 3000 column (Phenomenex) using 25 mM HEPES buffer, pH 7.5, containing 0.1% *b*-DM at a flow rate of 0.2 mL min^−1^ at 10 °C. Fractions were collected using an Agilent-1200 fraction collector. The column was calibrated using photosynthetic complexes of known size isolated from the cyanobacterium *Synechocystis* 6803: trimeric PSI (~1 MDa), dimeric PSII (~700 kDa), monomeric PSII lacking oxygen-evolving complex (~250 kDa) and the purified His-tagged CP43 assembly module (~90 kDa).

### Measurement of SOD activity

The SOD activity staining was performed as described in ref.^96^. Briefly, the strips from CN-gel were incubated with 0.1% nitroblue tetrazolium solution in dark for 20 min. After washing with water the gel was furter incubate with a solution of 28 μM riboflavin and 28 mM TEMED in 0.1 M potassium phosphate buffer (pH 7.0) in dark for 20 min. The washed gel was finally illuminated with a white-light box for 20 min to develop SOD activity bands. In order to identify different SOD species a gel-strip was incubated in 8 mM hydrogen peroxide prior to incubation with nitroblue tetrazolium to inhibit both ZnSOD and FeSOD activities. The ZnSOD activity only was inhibited by adding of 8 mM KCN into riboflavin solution.

## Electronic supplementary material

Supplementary Information
