# Supplementary material for: Extensive gain and loss of photosystem I subunits in chromerid algae, photosynthetic relatives of apicomplexans
Source: Sci Rep. 2017 Oct 16;7:13214. doi: 10.1038/s41598-017-13575-x (PMC5643376; doi:10.1038/s41598-017-13575-x)
Supplement: Supplementary file 1 — Supplementary Information [file 41598_2017_13575_MOESM1_ESM.pdf]

Supplementary material to:

Extensive gain and loss of photosystem I subunits in chromerid algae, photosynthetic relatives of apicomplexans

Roman Sobotka[1,3†], Heather J. Esson[2†], Peter Koník[1,3], Eliška Trsková[1,3], Lenka Moravcová[1,3], Aleš Horák[2,3], Petra Dufková[2] and Miroslav Oborník[1,2,3]

1. Centre Algatech, Institute of Microbiology, Czech Academy of Sciences, Třeboň, Czech Republic

2. Institute of Parasitology, Biological Centre, Czech Academy of Sciences, České Budějovice, Czech Republic

3. Faculty of Science, University of South Bohemia, České Budějovice, Czech Republic

[†]Authors contributed equally to the manuscript

Address for correspondence: sobotka@alga.cz; obornik@paru.cas.cz

This file includes Supplementary Materials and Methods, Supplementary Tables S1-S5, Supplementary Figures S1-S3, and References.

Supplementary Materials and Methods

Phylogenetic analysis of superoxide dismutases

A combination of BLAST and annotation searches were used to retrieve protein sequences from NCBI, JGI and other databases (see Table S2). Redundant sequences were removed from results and remaining sequences were aligned using MUSCLE (Edgar 2004) in AliView (Larsson 2014). The alignment (189 amino acids) was analysed using the RAxML online interface (Stamatakis et al. 2008) under the LG model with estimated gamma distribution and fixed base frequencies. Bootstrap support values were calculated from 100 replicates.

Bipartite transit sequence detection of iron superoxide dismutases in *Chromera velia* and *Vitrella brassicaformis*

Amino acid SOD sequences from *C. velia* and *V. brassicaformis* (Table S3) were probed for the presence of bipartite plastid targeting sequences using programs on the Center for Biological Sequence Analysis (CBSA) server (http://www.cbs.dtu.dk/services/), namely SignalP and TargetP (Nielsen et al. 1997; Emanuelsson et al. 2000; Petersen et al. 2011). Each sequence was submitted to the SignalP server to predict the presence and length of a signal peptide; when a signal peptide was present, the corresponding amino acids were removed from the initial sequence. The shortened sequence was then submitted to the TargetP server to determine whether an additional targeting sequence was present.

Additionally, sequences were queried using both the “full” (heterokonts) and “HECTAR^SEC” (all eukaryote) settings of HECTAR v.1.3 (http://webtools.sb-roscoff.fr/; Gschloessl et al. 2008).

Supplementary Tables and Figures

Supplementary Table S1: Thylakoid membrane complex proteins, accession numbers, and sources. CMGP: *Cyanidioschyzon merolae* Genome Project, http://merolae.biol.s.u-tokyo.ac.jp/ (Matsuzaki et al. 2004); CGP: *Cyanophora* Genome Project, http://cyanophora.rutgers.edu/cyanophora/home.php; KEGG: Kyoto Encyclopedia of Genes and Genomes, http://www.genome.jp/kegg/ (Kanehisa et al. 2015); JGI: Joint Genome Institute, http://genome.jgi.doe.gov/ (Curtis et al. 2012; Grigoriev et al. 2012; Nordberg et al. 2014); EuPathDB: Eukaryotic Pathogen Database Resources (Woo et al. 2015); TBestDB: Taxonomically Broad EST Database, http://tbestdb.bcm.umontreal.ca/.

Gene	Taxon	Accession number	Source
psaA	*Prochlorococcus marinus*	WP_011826946.1	NCBI
	Synechococcus elongatus	WP_011244354.1	NCBI
	Thermosynechococcus elongatus	NP_681520.1	NCBI
	Microcystis aeruginosa	WP_041804242.1	NCBI
	Anabaena variabilis	WP_011319188.1	NCBI
	Volvox carteri	ACY06050.1	GenBank
	Chlamydomonas reinhardtii	NP_958375.1	NCBI
	Ostreococcus tauri	YP_717254.1	NCBI
	Micromonas commoda	YP_002808608.1	NCBI
	Arabidopsis thaliana	NP_051059.1	NCBI
	Physcomitrella patens	NP_904202.1	NCBI
	Cyanidioschyzon merolae	CMV135C	CMGP
	Gracilaria tenuistipitata	YP_063614.2	NCBI
	Chondrus crispus	AAM62018.1	GenBank
	Cyanophora paradoxa	AAA81181.1	NCBI
	Glaucocystis nostochinearum	AAX82908.1	GenBank
	Guillardia theta	NP_050765.1	NCBI
	Emiliania huxleyi	YP_277311.1	NCBI
	Phaeodactylum tricornutum	YP_874359.1	NCBI
	Thalassiosira pseudonana	YP_874490.1	NCBI
	Fragilariopsis cylindrus	chloroplast scaffold_95	JGI
	Ectocarpus siliculosus	YP_003289256.1	NCBI
	Aureococcus anophagefferens	YP_003002086.1	NCBI
	Nannochloropsis gaditana	YP_007316990.1	NCBI
	Vitrella brassicaformis	ADJ66612.1	GenBank
	Chromera velia	YP_003795268.2	NCBI
	Bigelowiella natans	YP_778576.1	NCBI
	Paulinella chromatophora	YP_002049202.1	NCBI
	Euglena gracilis	NP_041906.1	NCBI
psaB	*Prochlorococcus marinus*	WP_011826945.1	NCBI
	Synechococcus elongatus	WP_011244355.1	NCBI
	Thermosynechococcus elongatus	NP_681521.1	NCBI

	Microcystis aeruginosa	WP_002770787.1	NCBI
	Anabaena variabilis	WP_011319189.1	NCBI
	Volvox carteri	ACY06053.1	GenBank
	Chlamydomonas reinhardtii	NP_958404.1	NCBI
	Ostreococcus tauri	YP_717253.1	NCBI
	Micromonas commoda	YP_002808607.1	NCBI
	Arabidopsis thaliana	NP_051058.1	NCBI
	Physcomitrella patens	NP_904203.1	NCBI
	Cyanidioschyzon merolae	CMV136C	CMGP
	Gracilaria tenuistipitata	YP_063613.1	NCBI
	Chondrus crispus	AAR30303.1	GenBank
	Cyanophora paradoxa	NP_043151.1	NCBI
	Glaucocystis nostochinearum	AAX82912.1	GenBank
	Guillardia theta	NP_050764.1	NCBI
	Emiliania huxleyi	YP_277312.1	NCBI
	Phaeodactylum tricornutum	YP_874358.1	NCBI
	Thalassiosira pseudonana	YP_874491.1	NCBI
	Fragilariopsis cylindrus	chloroplast scaffold_95	JGI
	Ectocarpus siliculosus	YP_003289257.1	NCBI
	Aureococcus anophagefferens	YP_003002087.1	NCBI
	Nannochloropsis gaditana	YP_007316991.1	NCBI
	Vitrella brassicaformis	ADJ66611.1	GenBank
	Chromera velia	YP_003795270.2	NCBI
	Bigelowiella natans	YP_778587.1	NCBI
	Paulinella chromatophora	YP_002049203.1	NCBI
	Euglena gracilis	NP_041907.1	NCBI
psaC	*Prochlorococcus marinus*	WP_011827179.1	NCBI
	Synechococcus elongatus	WP_011243298.1	NCBI
	Thermosynechococcus elongatus	NP_681803.1	NCBI
	Microcystis aeruginosa	WP_002734319.1	NCBI
	Anabaena variabilis	WP_010997613.1	NCBI
	Volvox carteri	ACY06004.1	GenBank
	Chlamydomonas reinhardtii	NP_958423.1	NCBI
	Ostreococcus tauri	YP_717214.1	NCBI
	Micromonas commoda	YP_002808655.1	NCBI
	Arabidopsis thaliana	NP_051110.1	NCBI
	Physcomitrella patens	NP_904239.1	NCBI
	Cyanidioschyzon merolae	CMV059C	CMGP
	Gracilaria tenuistipitata	YP_063690.1	NCBI
	Chondrus crispus	CHC_1075	KEGG
	Cyanophora paradoxa	NP_043270.1	NCBI
	Glaucocystis nostochinearum	-	
	Guillardia theta	NP_050694.1	NCBI
	Emiliania huxleyi	YP_277346.1	NCBI
	Phaeodactylum tricornutum	YP_874487.1	NCBI
	Thalassiosira pseudonana	YP_874576.1	NCBI
	Fragilariopsis cylindrus	chloroplast scaffold_95	JGI
	Ectocarpus siliculosus	YP_003289280.1	NCBI
	Aureococcus anophagefferens	YP_003002112.1	NCBI
	Nannochloropsis gaditana	YP_007317105.1	NCBI
	Vitrella brassicaformis	ADJ66600.1	GenBank
	Chromera velia	YP_003795326.1	NCBI
	Bigelowiella natans	YP_778580.1	NCBI
	Paulinella chromatophora	YP_002049117.1	NCBI
	Euglena gracilis	NP_041934.1	NCBI
psaD	*Prochlorococcus marinus*	WP_011826874.1	NCBI
	Synechococcus elongatus	WP_011377824.1	NCBI
	Thermosynechococcus elongatus	NP_682514.1	NCBI
	Microcystis aeruginosa	WP_002796771.1	NCBI
	Anabaena variabilis	WP_011321384.1	NCBI

	Volvox carteri	XP_002953316.1	NCBI
	Chlamydomonas reinhardtii	XP_001697722.1	NCBI
	Ostreococcus lucimarinus	XP_001421350.1	NCBI
	Micromonas commoda	XP_002504652.1	NCBI
	Arabidopsis thaliana	NP_171812.1	NCBI
	Physcomitrella patens	XP_001752431.1	NCBI
	Cyanidioschyzon merolae	CMV144C	CMGP
	Gracilaria tenuistipitata	YP_063618.1	NCBI
	Chondrus crispus	CHC_665	KEGG
	Cyanophora paradoxa	Q9T4W8.1	UniProtKB/Swiss-Prot
	Glaucocystis nostochinearum	EC125199.1	NCBI
	Guillardia theta	NP_050759.1	NCBI
	Emiliania huxleyi	YP_277398.1	NCBI
	Phaeodactylum tricornutum	YP_874394.1	NCBI
	Thalassiosira pseudonana	YP_874547.1	NCBI
	Fragilariopsis cylindrus	chloroplast scaffold_95	JGI
	Ectocarpus siliculosus	YP_003289238.1	NCBI
	Aureococcus anophagefferens	YP_003002042.1	NCBI
	Nannochloropsis gaditana	YP_007317065.1	NCBI
	Vitrella brassicaformis	ADJ66635.1	GenBank
	Chromera velia	Cvel_25206	EuPathDB
	Bigelowiella natans	AAP79147.1	GenBank
	Paulinella chromatophora	YP_002049251.1	NCBI
	Euglena gracilis	ELL00002449	TBestDB
psaE	*Prochlorococcus marinus*	ABM78910.1	GenBank
	Synechococcus elongatus	WP_011242545.1	NCBI
	Thermosynechococcus elongatus	NP_682357.1	NCBI
	Microcystis aeruginosa	WP_002797997.1	NCBI
	Anabaena variabilis	WP_011318110.1	NCBI
	Volvox carteri	XP_002948231.1	NCBI
	Chlamydomonas reinhardtii	XP_001702611.1	NCBI
	Ostreococcus tauri	XP_003075228.1	NCBI
	Micromonas commoda	XP_002502060.1	NCBI
	Arabidopsis thaliana	NP_567818.1	NCBI
	Physcomitrella patens	XP_001768221.1	NCBI
	Cyanidioschyzon merolae	CMV128C	CMGP
	Gracilaria tenuistipitata	YP_063572.1	NCBI
	Chondrus crispus	CHC_420	KEGG
	Cyanophora paradoxa	NP_043234.1	NCBI
	Glaucocystis nostochinearum	-	
	Guillardia theta	NP_050803.1	NCBI
	Emiliania huxleyi	EMIHUDRAFT_449561	KEGG
	Phaeodactylum tricornutum	YP_874428.1	NCBI
	Thalassiosira pseudonana	YP_874511.1	NCBI
	Fragilariopsis cylindrus	Fracy1|165544	JGI
	Ectocarpus siliculosus	YP_003289176.1	NCBI
	Aureococcus anophagefferens	XP_009036075.1	NCBI
	Nannochloropsis gaditana	YP_007317004.1	NCBI
	Vitrella brassicaformis	Vbra_21597	EuPathDB
	Chromera velia	Cvel_5788	EuPathDB
	Bigelowiella natans	AAP79148.1	GenBank
	Paulinella chromatophora	ACQ78178.1	GenBank
	Euglena gracilis	EGL00000033	TBestDB
psaF	*Prochlorococcus marinus*	WP_011130684.1	NCBI
	Synechococcus elongatus	WP_011242614.1	NCBI
	Thermosynechococcus elongatus	NP_683201.1	NCBI
	Microcystis aeruginosa	WP_002748906.1	NCBI
	Anabaena variabilis	WP_011318300.1	NCBI
	Volvox carteri	XP_002952500.1	NCBI

	Chlamydomonas reinhardtii	XP_001696798.1	NCBI
	Ostreococcus tauri	XP_003078706.1	NCBI
	Micromonas commoda	XP_002501874.1	NCBI
	Arabidopsis thaliana	NP_174418.1	NCBI
	Physcomitrella patens	PHYPADRAFT 151795 CDS	KEGG
	Cyanidioschyzon merolae	CMV201C	CMGP
	Gracilaria tenuistipitata	YP_063512.1	NCBI
	Chondrus crispus	CHC_25	KEGG
	Cyanophora paradoxa	NP_043153.1	NCBI
	Glaucocystis nostochinearum	-	
	Guillardia theta	NP_050714.1	NCBI
	Emiliania huxleyi	YP_277348.1	NCBI
	Phaeodactylum tricornutum	YP_874361.1	NCBI
	Thalassiosira pseudonana	YP_874562.1	NCBI
	Fragilariopsis cylindrus	chloroplast scaffold_95	JGI
	Ectocarpus siliculosus	YP_003289261.1	NCBI
	Aureococcus anophagefferens	XP_009039740.1	NCBI
	Nannochloropsis gaditana	YP_007317046.1	NCBI
	Vitrella brassicaformis	Vbra_1344	EuPathDB
	Chromera velia	Cvel_23764	EuPathDB
	Bigelowiella natans	AAP79177.1	GenBank
	Paulinella chromatophora	YP_002049385.1	NCBI
	Euglena gracilis	EGL00000854	TBestDB
psaI	*Prochlorococcus marinus*	ABM79080.1	GenBank
	Synechococcus elongatus	WP_011244070.1	NCBI
	Thermosynechococcus elongatus	NP_683195.1	NCBI
	Microcystis aeruginosa	WP_004162778.1	NCBI
	Anabaena variabilis	P23079.2	UniProtKB/Swiss-Prot
	Volvox carteri	XP_002951752.1	NCBI
	Chlamydomonas reinhardtii	XP_001703367.1	NCBI
	Ostreococcus tauri	YP_717255.1	NCBI
	Micromonas commoda	YP_002808609.1	NCBI
	Arabidopsis thaliana	NP_051069.1	NCBI
	Physcomitrella patens	NP_904192.1	NCBI
	Cyanidioschyzon merolae	CMV227C	CMGP
	Gracilaria tenuistipitata	YP_063702.1	NCBI
	Chondrus crispus	CHC_1150	KEGG
	Cyanophora paradoxa	NP_043182.1	NCBI
	Glaucocystis nostochinearum	-	
	Guillardia theta	NP_050719.1	NCBI
	Emiliania huxleyi	YP_277394.1	NCBI
	Phaeodactylum tricornutum	YP_874378.1	NCBI
	Thalassiosira pseudonana	YP_874541.1	NCBI
	Fragilariopsis cylindrus	Fracy1|267711	JGI
	Ectocarpus siliculosus	YP_003289284.1	NCBI
	Aureococcus anophagefferens	-	
	Nannochloropsis gaditana	AGI98707.1	GenBank
	Vitrella brassicaformis	-	
	Chromera velia	-	
	Bigelowiella natans	-	
	Paulinella chromatophora	YP_002049205.1	NCBI
	Euglena gracilis	-	
psaJ	*Prochlorococcus marinus*	Q7V658.1	UniProtKB/Swiss-Prot
	Synechococcus elongatus	WP_011242615.1	NCBI
	Thermosynechococcus elongatus	WP_011058244.1	NCBI
	Microcystis aeruginosa	BAG04552.1	GenBank

	Anabaena variabilis	P23080.3	UniProtKB/Swiss-Prot
	Volvox carteri	ACY05998.1	GenBank
	Chlamydomonas reinhardtii	NP_958417.1	NCBI
	Ostreococcus tauri	YP_717261.1	NCBI
	Micromonas commoda	YP_002808615.1	NCBI
	Arabidopsis thaliana	NP_051079.1	NCBI
	Physcomitrella patens	NP_904180.1	NCBI
	Cyanidioschyzon merolae	CMV202C	CMGP
	Gracilaria tenuistipitata	YP_063513.1	NCBI
	Chondrus crispus	CHC_30	KEGG
	Cyanophora paradoxa	NP_043152.1	NCBI
	Glaucocystis nostochinearum	-	
	Guillardia theta	NP_050713.1	NCBI
	Emiliania huxleyi	YP_277349.1	NCBI
	Phaeodactylum tricornutum	YP_874360.1	NCBI
	Thalassiosira pseudonana	YP_874563.1	NCBI
	Fragilariopsis cylindrus	chloroplast scaffold_95	JGI
	Ectocarpus siliculosus	YP_003289262.1	NCBI
	Aureococcus anophagefferens	YP_003002046.1	NCBI
	Nannochloropsis gaditana	YP_007317104.1	NCBI
	Vitrella brassicaformis	-	
	Chromera velia	-	
	Bigelowiella natans	YP_778596.1	NCBI
	Paulinella chromatophora	YP_002049384.1	NCBI
	Euglena gracilis	YP_002720092.1	NCBI
psaK	*Prochlorococcus marinus*	WP_011130065.1	NCBI
	Synechococcus elongatus	WP_011243420.1, WP_011242934.1	NCBI
	Thermosynechococcus elongatus	WP_011058106.1	NCBI
	Microcystis aeruginosa	WP_002734065.1	NCBI
	Anabaena variabilis	P23317.2	UniProtKB/Swiss-Prot
	Volvox carteri	XP_002946059.1	NCBI
	Chlamydomonas reinhardtii	XP_001697230.1	NCBI
	Ostreococcus lucimarinus	XP_001421973.1	NCBI
	Micromonas commoda	XP_002508869.1	NCBI
	Arabidopsis thaliana	NP_174327.1	NCBI
	Physcomitrella patens	PHYPADRAFT_28496	KEGG
	Cyanidioschyzon merolae	CMV055C	CMGP
	Gracilaria tenuistipitata	YP_063686.1	NCBI
	Chondrus crispus	CHC_1055	KEGG
	Cyanophora paradoxa	Contig53600	CGP
	Glaucocystis nostochinearum	-	
	Guillardia theta	NP_050695.1	NCBI
	Emiliania huxleyi	-	
	Phaeodactylum tricornutum	-	
	Thalassiosira pseudonana	-	
	Fragilariopsis cylindrus	-	
	Ectocarpus siliculosus	-	
	Aureococcus anophagefferens	-	
	Nannochloropsis gaditana	-	
	Vitrella brassicaformis	-	
	Chromera velia	-	
	Bigelowiella natans	-	
	Paulinella chromatophora	CP000815.1	GenBank
	Euglena gracilis	-	
psaL	*Prochlorococcus marinus*	ABM79081.1	GenBank
	Synechococcus elongatus	WP_011378415.1	NCBI
	Thermosynechococcus elongatus	NP_683194.1	NCBI

	Microcystis aeruginosa	WP_002757141.1	NCBI
	Anabaena variabilis	WP_011318298.1	NCBI
	Volvox carteri	XP_002948330.1	NCBI
	Chlamydomonas reinhardtii	XP_001691084.1	NCBI
	Ostreococcus tauri	XP_003074802.1	NCBI
	Micromonas commoda	XP_002504254.1	NCBI
	Arabidopsis thaliana	NP_193016.1	NCBI
	Physcomitrella patens	XP_001760296.1	NCBI
	Cyanidioschyzon merolae	CMV236C	CMGP
	Gracilaria tenuistipitata	YP_063666.1	NCBI
	Chondrus crispus	CHC_925	KEGG
	Cyanophora paradoxa	Contig53411	CGP
	Glaucocystis nostochinearum	EC123572.1	NCBI
	Guillardia theta	NP_050726.1	NCBI
	Emiliania huxleyi	YP_277352.1	NCBI
	Phaeodactylum tricornutum	YP_874366.1	NCBI
	Thalassiosira pseudonana	YP_874557.1	NCBI
	Fragilariopsis cylindrus	chloroplast scaffold_95	JGI
	Ectocarpus siliculosus	YP_003289277.1	NCBI
	Aureococcus anophagefferens	YP_003002022.1	NCBI
	Nannochloropsis gaditana	YP_007317052.1	NCBI
	Vitrella brassicaformis	Vbra_18375	EuPathDB
	Chromera velia	Cvel_23765	EuPathDB
	Bigelowiella natans	-	
	Paulinella chromatophora	YP_002049204.1	NCBI
	Euglena gracilis	-	
Ycf3	*Prochlorococcus marinus*	WP_011827224.1	NCBI
	Synechococcus elongatus	WP_011244091.1	NCBI
	Thermosynechococcus elongatus	BAC09077.1	GenBank
	Microcystis aeruginosa	WP_002760118.1	NCBI
	Anabaena variabilis	ABA23496.1	GenBank
	Volvox carteri	XP_002959772.1	NCBI
	Chlamydomonas reinhardtii	NP_958393.1	NCBI
	Ostreococcus tauri	YP_717224.1	NCBI
	Micromonas commoda	YP_002808646.1	NCBI
	Arabidopsis thaliana	NP_051060.2	NCBI
	Physcomitrella patens	NP_904201.1	NCBI
	Cyanidioschyzon merolae	CMV197C	CMGP
	Gracilaria tenuistipitata	YP_063637.1	NCBI
	Chondrus crispus	CHC_770	KEGG
	Cyanophora paradoxa	NP_043154.1	NCBI
	Glaucocystis nostochinearum	-	
	Guillardia theta	NP_050747.1	NCBI
	Emiliania huxleyi	YP_277373.1	NCBI
	Phaeodactylum tricornutum	YP_874408.1	NCBI
	Thalassiosira pseudonana	YP_874527.1	NCBI
	Fragilariopsis cylindrus	chloroplast scaffold_95	JGI
	Ectocarpus siliculosus	YP_003289268.1	NCBI
	Aureococcus anophagefferens	YP_003002115.1	NCBI
	Nannochloropsis gaditana	YP_007317077.1	NCBI
	Vitrella brassicaformis	ADJ66584.1	GenBank
	Chromera velia	YP_003795285.1	NCBI
	Bigelowiella natans	YP_778600.1	NCBI
	Paulinella chromatophora	YP_002048955.1	NCBI
	Euglena gracilis	ELL00003787	TBestDB
Ycf4	*Prochlorococcus marinus*	WP_011825507.1	NCBI
	Synechococcus elongatus	WP_011377665.1	NCBI
	Thermosynechococcus elongatus	NP_682178.1	NCBI
	Microcystis aeruginosa	WP_012267086.1	NCBI
	Anabaena variabilis	WP_011318084.1	NCBI

	Volvox carteri	ACY06031.1	GenBank
	Chlamydomonas reinhardtii	NP_958384.1	NCBI
	Ostreococcus tauri	XP_003079977.1	NCBI
	Micromonas commoda	XP_002507870.1	NCBI
	Arabidopsis thaliana	NP_051070.1	NCBI
	Physcomitrella patens	NP_904191.1	NCBI
	Cyanidioschyzon merolae	CMV233C	CMGP
	Gracilaria tenuistipitata	YP_063665.1	NCBI
	Chondrus crispus	CHC_915	KEGG
	Cyanophora paradoxa	NP_043147.1	NCBI
	Glaucocystis nostochinearum	-	
	Guillardia theta	NP_050696.1	NCBI
	Emiliania huxleyi	YP_277358.1	NCBI
	Phaeodactylum tricornutum	YP_874368.1	NCBI
	Thalassiosira pseudonana	YP_874555.1	NCBI
	Fragilariopsis cylindrus	chloroplast scaffold_95	JGI
	Ectocarpus siliculosus	YP_003289279.1	NCBI
	Aureococcus anophagefferens	YP_003002020.1	NCBI
	Nannochloropsis gaditana	YP_007317054.1	NCBI
	Vitrella brassicaformis	ADJ66577.1	GenBank
	Chromera velia	-	
	Bigelowiella natans	YP_778575.1	NCBI
	Paulinella chromatophora	YP_002048702.1	NCBI
	Euglena gracilis	NP_041899.1	NCBI
psbA	*Prochlorococcus marinus*	A2C6Q1.1	UniProtKB/Swiss-Prot
	Synechococcus elongatus	WP_011242480.1	NCBI
	Thermosynechococcus elongatus	NP_682633.1	NCBI
	Microcystis aeruginosa	WP_012264511.1	NCBI
	Anabaena variabilis	WP_011318925.1	NCBI
	Volvox carteri	ACY06027.1	GenBank
	Chlamydomonas reinhardtii	NP_958377.1	NCBI
	Ostreococcus tauri	YP_717233.1	NCBI
	Micromonas commoda	YP_002808606.1	NCBI
	Arabidopsis thaliana	NP_051039.1	NCBI
	Physcomitrella patens	NP_904209.1	NCBI
	Cyanidioschyzon merolae	CMV047C	CMGP
	Gracilaria tenuistipitata	YP_063531.1	NCBI
	Chondrus crispus	YP_007627313.1	NCBI
	Cyanophora paradoxa	NP_043238.1	NCBI
	Glaucocystis nostochinearum	AAX82914.1	GenBank
	Guillardia theta	NP_050699.1	NCBI
	Emiliania huxleyi	YP_277315.1	NCBI
	Phaeodactylum tricornutum	YP_874444.1	NCBI
	Thalassiosira pseudonana	YP_874574.1	NCBI
	Fragilariopsis cylindrus	Fracy1|164319	JGI
	Ectocarpus siliculosus	YP_003289145.1	NCBI
	Aureococcus anophagefferens	YP_003002124.1	NCBI
	Nannochloropsis gaditana	YP_007317069.1	NCBI
	Vitrella brassicaformis	ADJ66640.1	GenBank
	Chromera velia	YP_003795256.1	NCBI
	Bigelowiella natans	YP_778586.1	NCBI
	Paulinella chromatophora	ABH09268.1	GenBank
	Euglena gracilis	NP_041895.1	NCBI
psbB	*Prochlorococcus marinus*	CAE21840.1	GenBank
	Synechococcus elongatus	P31094.1	UniProtKB/Swiss-Prot
	Thermosynechococcus elongatus	NP_682320.1	NCBI
	Microcystis aeruginosa	WP_002757651.	NCBI
	Anabaena variabilis	ABA21130.1	GenBank

	Volvox carteri	ACY06046.1	GenBank
	Chlamydomonas reinhardtii	NP_958388.1	NCBI
	Ostreococcus tauri	Q0P3P8.2	UniProtKB/Swiss-Prot
	Micromonas commoda	YP_002808602.2	NCBI
	Arabidopsis thaliana	NP_051084.1	NCBI
	Physcomitrella patens	NP_904175.1	NCBI
	Cyanidioschyzon merolae	CMV124C	CMGP
	Gracilaria tenuistipitata	YP_063576.1	NCBI
	Chondrus crispus	CHC_440	KEGG
	Cyanophora paradoxa	NP_043167.1	NCBI
	Glaucocystis nostochinearum	-	
	Guillardia theta	NP_050799.1	NCBI
	Emiliania huxleyi	YP_277342.1	NCBI
	Phaeodactylum tricornutum	YP_874387.1	NCBI
	Thalassiosira pseudonana	YP_874507.1	NCBI
	Fragilariopsis cylindrus	chloroplast scaffold_95	JGI
	Ectocarpus siliculosus	YP_003289171.1	NCBI
	Aureococcus anophagefferens	YP_003002073.1	NCBI
	Nannochloropsis gaditana	YP_007317008.1	NCBI
	Vitrella brassicaformis	ADJ66608.1	GenBank
	Chromera velia	YP_003795264.2	NCBI
	Bigelowiella natans	Q06J24.1	UniProtKB/Swiss-Prot
	Paulinella chromatophora	YP_002049270.1	NCBI
	Euglena gracilis	NP_041946.1	NCBI
psbC	*Prochlorococcus marinus*	WP_011825504.1	NCBI
	Synechococcus elongatus	P11004.3	UniProtKB/Swiss-Prot
	Thermosynechococcus elongatus	NP_682421.1	NCBI
	Microcystis aeruginosa	WP_002739004.1	NCBI
	Anabaena variabilis	WP_011318086.1	NCBI
	Volvox carteri	ACY06003.1	GenBank
	Chlamydomonas reinhardtii	NP_958422.1	NCBI
	Ostreococcus tauri	Q0P3Q1.1	UniProtKB/Swiss-Prot
	Micromonas commoda	YP_002808605.1	NCBI
	Arabidopsis thaliana	NP_051055.1	NCBI
	Physcomitrella patens	Q6YXN9.1	UniProtKB/Swiss-Prot
	Cyanidioschyzon merolae	CMV082C	CMGP
	Gracilaria tenuistipitata	YP_063543.2	NCBI
	Chondrus crispus	YP_007627323.1	NCBI
	Cyanophora paradoxa	NP_043248.3	NCBI
	Glaucocystis nostochinearum	AAX82939.1	GenBank
	Guillardia theta	NP_050677.2	NCBI
	Emiliania huxleyi	YP_277371.1	NCBI
	Phaeodactylum tricornutum	YP_874376.2	NCBI
	Thalassiosira pseudonana	YP_874543.2	NCBI
	Fragilariopsis cylindrus	ABT17158.1	GenBank
	Ectocarpus siliculosus	YP_003289148.1	NCBI
	Aureococcus anophagefferens	YP_003002044.1	NCBI
	Nannochloropsis oculata	YP_008519669.1	NCBI
	Vitrella brassicaformis	ADJ66597.1	GenBank
	Chromera velia	YP_003795274.1	NCBI
	Bigelowiella natans	Q06J66.1	UniProtKB/Swiss-Prot
	Paulinella chromatophora	YP_002048704.1	NCBI
	Euglena gracilis	NP_041892.1	NCBI
psbD	*Prochlorococcus marinus*	WP_012195785.1	NCBI

	Synechococcus elongatus	WP_011243185.1	NCBI
	Thermosynechococcus elongatus	WP_011056308.1	NCBI
	Microcystis aeruginosa	WP_002737445.1	NCBI
	Anabaena variabilis	WP_011318085.1	NCBI
	Volvox carteri	ACI31257.1	GenBank
	Chlamydomonas reinhardtii	NP_958420.1	NCBI
	Ostreococcus tauri	YP_717205.1	NCBI
	Micromonas commoda	YP_002808604.1	NCBI
	Arabidopsis thaliana	NP_051054.1	NCBI
	Physcomitrella patens	NP_904207.1	NCBI
	Cyanidioschyzon merolae	CMV081C	CMGP
	Gracilaria tenuistipitata	YP_063542.1	NCBI
	Chondrus crispus	CHC_205	KEGG
	Cyanophora paradoxa	NP_043247.1	NCBI
	Glaucocystis nostochinearum	-	
	Guillardia theta	NP_050678.1	NCBI
	Emiliania huxleyi	YP_277372.1	NCBI
	Phaeodactylum tricornutum	YP_874377.1	NCBI
	Thalassiosira pseudonana	YP_874542.1	NCBI
	Fragilariopsis cylindrus	chloroplast scaffold_95	JGI
	Ectocarpus siliculosus	YP_003289147.1	NCBI
	Aureococcus anophagefferens	YP_003002045.1	NCBI
	Nannochloropsis gaditana	YP_007317039.1	NCBI
	Vitrella brassicaformis	YP_003795459.1	NCBI
	Chromera velia	YP_003795275.1	NCBI
	Bigelowiella natans	YP_778560.1	NCBI
	Paulinella chromatophora	YP_002048703.1	NCBI
	Euglena gracilis	P31557.1	UniProtKB/Swiss-Prot
psbE	*Prochlorococcus marinus*	WP_011131262.1	NCBI
	Synechococcus elongatus	WP_011242685.1	NCBI
	Thermosynechococcus elongatus	NP_682331.1	NCBI
	Microcystis aeruginosa	WP_002749269.1	NCBI
	Anabaena variabilis	ABA21477.1	NCBI
	Volvox carteri	ACY06033.1	GenBank
	Chlamydomonas reinhardtii	NP_958396.1	NCBI
	Ostreococcus tauri	YP_717219.1	NCBI
	Micromonas commoda	YP_002808650.1	NCBI
	Arabidopsis thaliana	NP_051076.1	NCBI
	Physcomitrella patens	NP_904183.1	NCBI
	Cyanidioschyzon merolae	CMV231C	CMGP
	Gracilaria tenuistipitata	YP_063706.1	NCBI
	Chondrus crispus	CHC_1170	KEGG
	Cyanophora paradoxa	NP_043178.1	NCBI
	Glaucocystis nostochinearum	-	
	Guillardia theta	NP_050723.1	NCBI
	Emiliania huxleyi	YP_277387.1	NCBI
	Phaeodactylum tricornutum	YP_874369.1	NCBI
	Thalassiosira pseudonana	YP_874554.1	NCBI
	Fragilariopsis cylindrus	chloroplast scaffold_95	JGI
	Ectocarpus siliculosus	YP_003289288.1	NCBI
	Aureococcus anophagefferens	YP_003002085.1	NCBI
	Nannochloropsis gaditana	YP_007317055.1	NCBI
	Vitrella brassicaformis	ADJ66578.1	GenBank
	Chromera velia	YP_003795269.1	NCBI
	Bigelowiella natans	YP_778616.1	NCBI
	Paulinella chromatophora	YP_002049138.1	NCBI
	Euglena gracilis	NP_041908.1	NCBI
psbF	*Prochlorococcus marinus*	ABM79261.1	GenBank
	Synechococcus elongatus	WP_011242686.1	NCBI

	Thermosynechococcus elongatus	NP_682332.1	NCBI
	Microcystis aeruginosa	WP_002736175.1	NCBI
	Anabaena variabilis	Q3MC10.1	UniProtKB/Swiss-Prot
	Volvox carteri	ACY06044.1	GenBank
	Chlamydomonas reinhardtii	NP_958399.1	NCBI
	Ostreococcus tauri	YP_717218.1	NCBI
	Micromonas commoda	YP_002808651.1	NCBI
	Arabidopsis thaliana	NP_051075.1	NCBI
	Physcomitrella patens	NP_904184.1	NCBI
	Cyanidioschyzon merolae	CMV230C	CMGP
	Gracilaria tenuistipitata	YP_063705.1	NCBI
	Chondrus crispus	CHC_1165	KEGG
	Cyanophora paradoxa	NP_043179.1	NCBI
	Glaucocystis nostochinearum	-	
	Guillardia theta	NP_050722.1	NCBI
	Emiliania huxleyi	YP_277386.1	NCBI
	Phaeodactylum tricornutum	YP_874370.1	NCBI
	Thalassiosira pseudonana	YP_874553.1	NCBI
	Fragilariopsis cylindrus	chloroplast scaffold_95	JGI
	Ectocarpus siliculosus	YP_003289287.1	NCBI
	Aureococcus anophagefferens	YP_003002084.1	NCBI
	Nannochloropsis gaditana	YP_007317056.1	NCBI
	Vitrella brassicaformis	ADJ66579.1	GenBank
	Chromera velia	Cvel_23342	EuPathDB
	Bigelowiella natans	YP_778615.1	NCBI
	Paulinella chromatophora	YP_002049137.1	NCBI
	Euglena gracilis	NP_041909.1	NCBI
psbH	*Prochlorococcus marinus*	ABM79193.1	GenBank
	Synechococcus elongatus	WP_011243600.1	NCBI
	Thermosynechococcus elongatus	NP_682176.1	NCBI
	Microcystis aeruginosa	B0JGF6.1	UniProtKB/Swiss-Prot
	Anabaena variabilis	Q3M4N8.1	NCBI
	Volvox carteri	XP_002959711.1	NCBI
	Chlamydomonas reinhardtii	NP_958385.1	NCBI
	Ostreococcus tauri	YP_717221.1	NCBI
	Micromonas commoda	YP_002808649.1	NCBI
	Arabidopsis thaliana	NP_051087.1	NCBI
	Physcomitrella patens	Q6YXN1.3	UniProtKB/Swiss-Prot
	Cyanidioschyzon merolae	CMV127C	CMGP
	Gracilaria tenuistipitata	YP_063573.1	NCBI
	Chondrus crispus	CHC_425	KEGG
	Cyanophora paradoxa	NP_043169.1	NCBI
	Glaucocystis nostochinearum	-	
	Guillardia theta	NP_050802.1	NCBI
	Emiliania huxleyi	YP_277345.1	NCBI
	Phaeodactylum tricornutum	YP_874384.1	NCBI
	Thalassiosira pseudonana	YP_874510.1	NCBI
	Fragilariopsis cylindrus	chloroplast scaffold_95	JGI
	Ectocarpus siliculosus	YP_003289174.1	NCBI
	Aureococcus anophagefferens	YP_003002076.1	NCBI
	Nannochloropsis gaditana	YP_007317005.1	NCBI
	Vitrella brassicaformis	ADJ66582.1	GenBank
	Chromera velia	YP_003795330.1	NCBI
	Bigelowiella natans	YP_778606.1	NCBI
	Paulinella chromatophora	YP_002049166.1	NCBI
	Euglena gracilis	NP_041944.1	NCBI
psbI	*Prochlorococcus marinus*	ABM79196.1	GenBank

	Synechococcus elongatus	P17747.1	UniProtKB/Swiss-Prot
	Thermosynechococcus elongatus	NP_681865.1	NCBI
	Microcystis aeruginosa	B0JX58.1	UniProtKB/Swiss-Prot
	Anabaena variabilis	ABA22611.1	GenBank
	Volvox carteri	ACY06059.1	GenBank
	Chlamydomonas reinhardtii	P59763.1	UniProtKB/Swiss-Prot
	Ostreococcus tauri	YP_717260.1	NCBI
	Micromonas commoda	YP_002808614.1	NCBI
	Arabidopsis thaliana	NP_051043.1	NCBI
	Physcomitrella patens	NP_904213.2	NCBI
	Cyanidioschyzon merolae	CMV240C	CMGP
	Gracilaria tenuistipitata	YP_063658.1	NCBI
	Chondrus crispus	CHC_880	KEGG
	Cyanophora paradoxa	NP_043236.1	NCBI
	Glaucocystis nostochinearum	-	
	Guillardia theta	NP_050728.1	NCBI
	Emiliania huxleyi	YP_277320.1	NCBI
	Phaeodactylum tricornutum	YP_874362.1	NCBI
	Thalassiosira pseudonana	YP_874561.1	NCBI
	Fragilariopsis cylindrus	chloroplast scaffold_95	JGI
	Ectocarpus siliculosus	YP_003289228.1	NCBI
	Aureococcus anophagefferens	YP_003002094.1	NCBI
	Nannochloropsis gaditana	YP_007317066.1	NCBI
	Vitrella brassicaformis	ADJ66610.1	GenBank
	Chromera velia	-	
	Bigelowiella natans	Q06J30.1	UniProtKB/Swiss-Prot
	Paulinella chromatophora	YP_002049165.1	NCBI
	Euglena gracilis	NP_041904.1	NCBI
psbJ	*Prochlorococcus marinus*	ABM79263.1	GenBank
	Synechococcus elongatus	AAM82677.1	GenBank
	Thermosynechococcus elongatus	NP_682334.1	NCBI
	Microcystis aeruginosa	B0JLU8.1	UniProtKB/Swiss-Prot
	Anabaena variabilis	Q3MC12.2	UniProtKB/Swiss-Prot
	Volvox carteri	ACY06000.1	GenBank
	Chlamydomonas reinhardtii	NP_958419.1	NCBI
	Ostreococcus tauri	YP_717216.1	NCBI
	Micromonas commoda	YP_002808653.1	NCBI
	Arabidopsis thaliana	NP_051073.1	NCBI
	Physcomitrella patens	Q6YXL6.1	UniProtKB/Swiss-Prot
	Cyanidioschyzon merolae	CMV228C	CMGP
	Gracilaria tenuistipitata	YP_063703.1	NCBI
	Chondrus crispus	CHC_1155	KEGG
	Cyanophora paradoxa	NP_043181.1	NCBI
	Glaucocystis nostochinearum	-	
	Guillardia theta	NP_050720.1	NCBI
	Emiliania huxleyi	YP_277384.1	NCBI
	Phaeodactylum tricornutum	YP_874372.1	NCBI
	Thalassiosira pseudonana	YP_874551.1	NCBI
	Fragilariopsis cylindrus	-	
	Ectocarpus siliculosus	YP_003289285.1	NCBI
	Aureococcus anophagefferens	YP_003002082.1	NCBI
	Nannochloropsis gaditana	YP_007317057.1	NCBI
	Vitrella brassicaformis	ADJ66643.1	GenBank

	Chromera velia	YP_003795286.1	NCBI
	Bigelowiella natans	YP_778613.1	NCBI
	Paulinella chromatophora	YP_002049135.1	NCBI
	Euglena gracilis	NP_041911.1	NCBI
psbK	*Prochlorococcus marinus*	ABM79222.1	GenBank
	Synechococcus elongatus	P14089.1	UniProtKB/Swiss-Prot
	Thermosynechococcus elongatus	NP_680967.1	NCBI
	Microcystis aeruginosa	B0JSW8.1	UniProtKB/Swiss-Prot
	Anabaena variabilis	WP_010995059.1	NCBI
	Volvox carteri	ACY06006.1	GenBank
	Chlamydomonas reinhardtii	NP_958361.1	NCBI
	Ostreococcus tauri	YP_717259.1	NCBI
	Micromonas commoda	YP_002808613.1	NCBI
	Arabidopsis thaliana	NP_051042.1	NCBI
	Physcomitrella patens	Q6YXN4.1	UniProtKB/Swiss-Prot
	Cyanidioschyzon merolae	CMV140C	CMGP
	Gracilaria tenuistipitata	YP_063610.1	NCBI
	Chondrus crispus	CHC_615	KEGG
	Cyanophora paradoxa	NP_043249.1	NCBI
	Glaucocystis nostochinearum	-	
	Guillardia theta	NP_050761.1	NCBI
	Emiliania huxleyi	YP_277340.1	NCBI
	Phaeodactylum tricornutum	YP_874379.1	NCBI
	Thalassiosira pseudonana	YP_874540.1	NCBI
	Fragilariopsis cylindrus	chloroplast scaffold_95	JGI
	Ectocarpus siliculosus	YP_003289211.1	NCBI
	Aureococcus anophagefferens	YP_003002023.1	NCBI
	Nannochloropsis gaditana	YP_007316994.1	NCBI
	Vitrella brassicaformis	ADJ66603.1	GenBank
	Chromera velia	YP_003795283.1	NCBI
	Bigelowiella natans	YP_778611.1	NCBI
	Paulinella chromatophora	YP_002049151.1	NCBI
	Euglena gracilis	NP_041898.1	NCBI
psbL	*Prochlorococcus marinus*	WP_011827106.1	NCBI
	Synechococcus elongatus	WP_011377901.1	NCBI
	Thermosynechococcus elongatus	NP_682333.1	NCBI
	Microcystis aeruginosa	BAG03123.1	GenBank
	Anabaena variabilis	Q3MC11.1	UniProtKB/Swiss-Prot
	Volvox carteri	ACY06043.1	GenBank
	Chlamydomonas reinhardtii	NP_958400.2	NCBI
	Ostreococcus tauri	YP_717217.1	NCBI
	Micromonas commoda	YP_002808652.1	NCBI
	Arabidopsis thaliana	NP_051074.1	NCBI
	Physcomitrella patens	NP_904185.1	NCBI
	Cyanidioschyzon merolae	CMV229C	CMGP
	Gracilaria tenuistipitata	YP_063704.1	NCBI
	Chondrus crispus	CHC_1160	KEGG
	Cyanophora paradoxa	NP_043180.1	NCBI
	Glaucocystis nostochinearum	-	
	Guillardia theta	NP_050721.1	NCBI
	Emiliania huxleyi	YP_277385.1	NCBI
	Phaeodactylum tricornutum	YP_874371.1	NCBI
	Thalassiosira pseudonana	YP_874552.1	NCBI
	Fragilariopsis cylindrus	chloroplast scaffold_95	JGI
	Ectocarpus siliculosus	YP_003289286.1	NCBI
	Aureococcus anophagefferens	YP_003002083.1	NCBI

	Nannochloropsis salina	YP_008519855.1	NCBI
	Vitrella brassicaformis	Vbra_11908	EuPathDB
	Chromera velia	Cvel_21989	EuPathDB
	Bigelowiella natans	YP_778614.1	NCBI
	Paulinella chromatophora	B1X4Q7.1	UniProtKB/Swiss-Prot
	Euglena gracilis	NP_041910.1	NCBI
psbM	*Prochlorococcus marinus*	WP_011131030.1	NCBI
	Synechococcus elongatus	WP_011243143.1	NCBI
	Thermosynechococcus elongatus	NP_682842.1	NCBI
	Microcystis aeruginosa	B0JYB0.1	UniProtKB/Swiss-Prot
	Anabaena cylindrica	AFZ59679.1	NCBI
	Volvox carteri	ACY06038.1	GenBank
	Chlamydomonas reinhardtii	NP_958382.1	NCBI
	Ostreococcus tauri	XP_003082196.1	NCBI
	Micromonas commoda	XP_002506594.1	NCBI
	Arabidopsis thaliana	NP_051053.1	NCBI
	Physcomitrella patens	NP_904167.1	NCBI
	Cyanidioschyzon merolae	CMT182C	CMGP
	Gracilaria tenuistipitata	HS979039.1	GenBank
	Chondrus crispus	-	
	Cyanophora paradoxa	NP_043250.1	NCBI
	Glaucocystis nostochinearum	-	
	Guillardia theta	XP_005821709.1	NCBI
	Emiliania huxleyi	XP_005793513.1	NCBI
	Phaeodactylum tricornutum	AAO43193.1	GenBank
	Thalassiosira pseudonana	XP_002288939.1	NCBI
	Fragilariopsis cylindrus	Fracy1_172395	JGI
	Ectocarpus siliculosus	CBJ33411.1	GenBank
	Aureococcus anophagefferens	-	
	Nannochloropsis gaditana	-	
	Vitrella brassicaformis	Vbra_3546	EuPathDB
	Chromera velia	-	
	Bigelowiella natans	YP_778579.1	NCBI
	Paulinella chromatophora	YP_002049272.1	NCBI
	Euglena gracilis	ADJ93799.1	GenBank
psbN	*Prochlorococcus marinus*	WP_011827045.1	NCBI
	Synechococcus elongatus	WP_011243601.1	NCBI
	Thermosynechococcus elongatus	NP_682177.1	NCBI
	Microcystis aeruginosa	WP_002799553.1	NCBI
	Anabaena variabilis	Q3M4N7.1	UniProtKB/Swiss-Prot
	Volvox carteri	ACY06048.1	GenBank
	Chlamydomonas reinhardtii	NP_958386.1	NCBI
	Ostreococcus tauri	YP_717222.1	NCBI
	Micromonas commoda	YP_002808648.1	NCBI
	Arabidopsis thaliana	NP_051086.1	NCBI
	Physcomitrella patens	NP_904173.1	NCBI
	Cyanidioschyzon merolae	CMV126C	CMGP
	Gracilaria tenuistipitata	YP_063574.1	NCBI
	Chondrus crispus	CHC_430	KEGG
	Cyanophora paradoxa	NP_043165.1	NCBI
	Glaucocystis nostochinearum	-	
	Guillardia theta	NP_050801.1	NCBI
	Emiliania huxleyi	YP_277344.1	NCBI
	Phaeodactylum tricornutum	YP_874385.1	NCBI
	Thalassiosira pseudonana	YP_874509.1	NCBI
	Fragilariopsis cylindrus	chloroplast scaffold_95	JGI
	Ectocarpus siliculosus	YP_003289173.1	NCBI

	Aureococcus anophagefferens	YP_003002075.1	NCBI
	Nannochloropsis gaditana	YP_007317006.1	NCBI
	Vitrella brassicaformis	ADJ66581.1	GenBank
	Chromera velia	YP_003795296.1	NCBI
	Bigelowiella natans	YP_778605.1	NCBI
	Paulinella chromatophora	C02166	Nowack *et al.* 2011
	Euglena gracilis	NP_041943.1	NCBI
psbO	*Prochlorococcus marinus*	ABM79118.1	GenBank
	Synechococcus elongatus	P11472.1	UniProtKB/Swiss-Prot
	Thermosynechococcus elongatus	NP_681234.1	NCBI
	Microcystis aeruginosa	WP_004162736.1	NCBI
	Anabaena variabilis	WP_011318645.1	NCBI
	Volvox carteri	AAD55562.1	GenBank
	Chlamydomonas reinhardtii	XP_001694699.1	NCBI
	Ostreococcus tauri	ADC54905.1	GenBank
	Micromonas commoda	XP_002505809.1	NCBI
	Arabidopsis thaliana	NP_201458.1	NCBI
	Physcomitrella patens	XP_001763258.1	NCBI
	Cyanidioschyzon merolae	CMI290C	CMGP
	Gracilaria tenuistipitata	HS978096.1	NCBI EST
	Chondrus crispus	XP_005713751.1	NCBI
	Cyanophora paradoxa	CAH04962.1	GenBank
	Glaucocystis nostochinearum	EC124716.1	NCBI
	Guillardia theta	XP_005824851.1	NCBI
	Emiliania huxleyi	XP_005761454.1	NCBI
	Phaeodactylum tricornutum	XP_002180309.1	NCBI
	Thalassiosira pseudonana	XP_002291225.1	NCBI
	Fragilariopsis cylindrus	167955	JGI
	Ectocarpus siliculosus	CBN78902.1	GenBank
	Aureococcus anophagefferens	XP_009033884.1	NCBI
	Nannochloropsis gaditana	EWM24379.1	GenBank
	Vitrella brassicaformis	Vbra_7099	EuPathDB
	Chromera velia	Cvel_27124	EuPathDB
	Bigelowiella natans	AAP79149.1	GenBank
	Paulinella chromatophora	YP_002049183.1	NCBI
	Euglena gracilis	BAA03529.2	GenBank
psbP/cyanoP	*Prochlorococcus marinus*	WP_063420050.1	NCBI
	Synechococcus elongatus	WP_011242821.1	NCBI
	Thermosynechococcus elongatus	WP_011057910.1	NCBI
	Microcystis aeruginosa	WP_046662781.1	NCBI
	Anabaena variabilis	ABA20458.1	GenBank
	Volvox carteri	XP_002950336.1	NCBI
	Chlamydomonas reinhardtii	XP_001693241.1	NCBI
	Ostreococcus tauri	XP_003075035.1	NCBI
	Micromonas pusilla	XP_003056466.1	NCBI
	Arabidopsis thaliana	NP_565149.2	NCBI
	Physcomitrella patens	XP_001754475.1	NCBI
	Cyanidioschyzon merolae	XP_005537400.1	NCBI
	Gracilaria tenuistipitata	-	
	Chondrus crispus	XP_005711844.1	NCBI
	Cyanophora paradoxa	Contig6796/CGP	
	Glaucocystis nostochinearum	-	
	Guillardia theta	EKX33663.1	GenBank
	Emiliania huxleyi	-	
	Phaeodactylum tricornutum	XP_002177783.1	NCBI
	Thalassiosira pseudonana	EJK63943.1	GenBank
	Fragilariopsis cylindrus	Fracy1|142679	
	Ectocarpus siliculosus	CBN79490.1	GenBank

	Aureococcus anophagefferens	-	
	Nannochloropsis gaditana	EWM25422.1	GenBank
	Vitrella brassicaformis	Vbra_12477 OR Vbra_15129	EuPathDB
	Chromera velia	Cvel_20184 OR Cvel_22018	EuPathDB
	Bigelowiella natans	AAP79210.1	GenBank
	Paulinella chromatophora	YP_002049423.1	NCBI
	Euglena gracilis	P83687.1	UniProtKB/Swiss-Prot
psbQ	*Prochlorococcus marinus*	-	
	Synechococcus elongatus	WP_011244722.1	NCBI
	Thermosynechococcus elongatus	NP_682847.1	NCBI
	Microcystis aeruginosa	WP_002795844.1	NCBI
	Anabaena variabilis	ABA24633.1	NCBI
	Volvox carteri	XP_002947155.1	NCBI
	Chlamydomonas reinhardtii	XP_001701331.1	NCBI
	Ostreococcus tauri	XP_003083616.1	NCBI
	Micromonas commoda	XP_002506526.1	NCBI
	Arabidopsis thaliana	NP_193860.1	NCBI
	Physcomitrella patens	XP_001760196.1	NCBI
	Cyanidioschyzon merolae	CMC133C	CMGP
	Gracilaria tenuistipitata	HS979963.1	GenBank
	Chondrus crispus	CHC_T00001103001	KEGG
	Cyanophora paradoxa	-	
	Glaucocystis nostochinearum	-	
	Guillardia theta	XP_005821040.1	NCBI
	Emiliania huxleyi	XP_005763993.1	NCBI
	Phaeodactylum tricornutum	XP_002180307.1	NCBI
	Thalassiosira pseudonana	XP_002286571.1	NCBI
	Fragilariopsis cylindrus	Fracy1|206783	JGI
	Ectocarpus siliculosus	CBN75463.1	GenBank
	Aureococcus anophagefferens	XP_009040815.1	NCBI
	Nannochloropsis gaditana	XP_005854604.1	NCBI
	Vitrella brassicaformis	Vbra_1771	EuPathDB
	Chromera velia	Cvel_18330	EuPathDB
	Bigelowiella natans	AAP79208.1	GenBank
	Paulinella chromatophora	YP_002049043.1	NCBI
	Euglena gracilis	ELL00000039	TBestDB
psbT	*Prochlorococcus marinus*	WP_011131033.1	NCBI
	Synechococcus elongatus	WP_011243146.1	NCBI
	Thermosynechococcus elongatus	NP_682321.1	NCBI
	Microcystis aeruginosa	B0JTA8.1	UniProtKB/Swiss-Prot
	Anabaena variabilis	Q3MD07.1	UniProtKB/Swiss-Prot
	Volvox carteri	ACY06047.1	GenBank
	Chlamydomonas reinhardtii	NP_958387.1	NCBI
	Ostreococcus tauri	YP_717206.1	NCBI
	Micromonas commoda	YP_002808603.1	NCBI
	Arabidopsis thaliana	NP_051085.1	NCBI
	Physcomitrella patens	NP_904174.1	NCBI
	Cyanidioschyzon merolae	CMV125C	CMGP
	Gracilaria tenuistipitata	YP_063575.1	NCBI
	Chondrus crispus	YP_007627356.1	KEGG
	Cyanophora paradoxa	NP_043166.1	NCBI
	Glaucocystis nostochinearum	-	
	Guillardia theta	NP_050800.1	NCBI
	Emiliania huxleyi	YP_277343.1	NCBI
	Phaeodactylum tricornutum	YP_874386.1	NCBI

	Thalassiosira pseudonana	YP_874508.1	NCBI
	Fragilariopsis cylindrus	chloroplast scaffold_95	JGI
	Ectocarpus siliculosus	YP_003289172.1	NCBI
	Aureococcus anophagefferens	YP_003002074.1	NCBI
	Nannochloropsis gaditana	YP_007317007.1	NCBI
	Vitrella brassicaformis	ADJ66642.1	GenBank
	Chromera velia	YP_003795325.1	NCBI
	Bigelowiella natans	YP_778604.1	NCBI
	Paulinella chromatophora	YP_002049269.1	NCBI
	Euglena gracilis	NP_041945.1	NCBI
psbU	*Prochlorococcus marinus*	A2C664.1	UniProtKB/Swiss-Prot
	Synechococcus elongatus	WP_011244523.1	NCBI
	Thermosynechococcus elongatus	WP_011058241.1	NCBI
	Microcystis aeruginosa	WP_012266475.1	NCBI
	Anabaena variabilis	ABA20259.1	GenBank
	Volvox carteri	-	
	Chlamydomonas reinhardtii	-	
	Ostreococcus	-	
	Micromonas commoda	-	
	Arabidopsis thaliana	-	
	Physcomitrella patens	-	
	Cyanidioschyzon merolae	CMI248C	CMGP
	Gracilaria tenuistipitata	HS981649.1	GenBank
	Chondrus crispus	XP_005713750.1	KEGG
	Cyanophora paradoxa	Q5CC96.1	UniProtKB/Swiss-Prot
	Glaucocystis nostochinearum	-	
	Guillardia theta	XP_005833081.1	NCBI
	Emiliania huxleyi	XP_005757039.1	NCBI
	Phaeodactylum tricornutum	Q84XB6.1	UniProtKB/Swiss-Prot
	Thalassiosira pseudonana	XP_002288025.1	NCBI
	Fragilariopsis cylindrus	GW070000	NCBI EST
	Ectocarpus siliculosus	CBJ28280.1	GenBank
	Aureococcus anophagefferens	XP_009040947.1	NCBI
	Nannochloropsis gaditana	EWM24702.1	GenBank
	Vitrella brassicaformis	Vbra_15278, Vbra_6554	EuPathDB
	Chromera velia	Cvel_3244	EuPathDB
	Bigelowiella natans	-	
	Paulinella chromatophora	YP_002048936.1	NCBI
	Euglena gracilis	-	
psbV	*Prochlorococcus marinus*		UniProtKB/Swiss-Prot
	Synechococcus elongatus	ABB58040.1	GenBank
	Thermosynechococcus elongatus	WP_011057125.1	NCBI
	Microcystis aeruginosa	WP_012264644.1	NCBI
	Anabaena variabilis	Q3M9H7.1	UniProtKB/Swiss-Prot
	Volvox carteri	-	
	Chlamydomonas reinhardtii	-	
	Ostreococcus	-	
	Micromonas commoda	-	
	Arabidopsis thaliana	-	
	Physcomitrella patens	-	
	Cyanidioschyzon merolae	CMV208C	CMGP
	Gracilaria tenuistipitata	YP_063518.1	NCBI
	Chondrus crispus	CHC_60	KEGG
	Cyanophora paradoxa	NP_043260.1	NCBI

	Glaucocystis nostochinearum	-	
	Guillardia theta	NP_050711.1	NCBI
	Emiliania huxleyi	YP_277399.1	NCBI
	Phaeodactylum tricornutum	YP_874401.1	NCBI
	Thalassiosira pseudonana	YP_874494.1	NCBI
	Fragilariopsis cylindrus	chloroplast scaffold_95	JGI
	Ectocarpus siliculosus	YP_003289224.1	NCBI
	Aureococcus anophagefferens	YP_003002079.1	NCBI
	Nannochloropsis gaditana	YP_007316988.1	NCBI
	Vitrella brassicaformis	ADJ66606.1	GenBank
	Chromera velia	YP_003795282.1	NCBI
	Bigelowiella natans	-	
	Paulinella chromatophora	YP_002049328.1	NCBI
	Euglena gracilis	-	
psbX	*Prochlorococcus marinus*	WP_011130957.1	NCBI
	Synechococcus elongatus	WP_011244389.1	NCBI
	Thermosynechococcus elongatus	NP_682803.1	NCBI
	Microcystis aeruginosa	BAG04955.1	GenBank
	Anabaena variabilis	Q3MFU4.1	UniProtKB/Swiss-Prot
	Volvox carteri	XP_002953587.1	NCBI
	Chlamydomonas reinhardtii	XP_001701704.1	NCBI
	Ostreococcus lucimarinus	XP_001416212.1	NCBI
	Micromonas commoda	XP_002507848.1	NCBI
	Arabidopsis thaliana	NP_565335.1	NCBI
	Physcomitrella patens	XP_001779033.1	NCBI
	Cyanidioschyzon merolae	CMV206C	CMGP
	Gracilaria tenuistipitata	YP_063516.1	NCBI
	Chondrus crispus	CHC_50	KEGG
	Cyanophora paradoxa	NP_043252.1	NCBI
	Glaucocystis nostochinearum	-	
	Guillardia theta	NP_050712.1	NCBI
	Emiliania huxleyi	Q4G3D8.1	UniProtKB/Swiss-Prot
	Phaeodactylum tricornutum	YP_874399.1	NCBI
	Thalassiosira pseudonana	YP_874496.1	NCBI
	Fragilariopsis cylindrus	-	
	Ectocarpus siliculosus	YP_003289222.1	NCBI
	Aureococcus anophagefferens	YP_003002078.1	NCBI
	Nannochloropsis gaditana	-	
	Vitrella brassicaformis	-	
	Chromera velia	-	
	Bigelowiella natans	Bigna1|86445	JGI
	Paulinella chromatophora	YP_002049300.1	NCBI
	Euglena gracilis	-	
psbY	*Prochlorococcus marinus*	ABM77752.1	GenBank
	Synechococcus elongatus	WP_011244443.1	NCBI
	Thermosynechococcus elongatus	WP_011056680.1	NCBI
	Microcystis aeruginosa	WP_012265998.1	NCBI
	Anabaena variabilis	Q3M6U8.1	UniProtKB/Swiss-Prot
	Volvox carteri	XP_002960025.1	NCBI
	Chlamydomonas reinhardtii	XP_001698338.1	NCBI
	Ostreococcus tauri	XP_003082206.1	NCBI
	Micromonas commoda	XP_002502801.1	NCBI
	Arabidopsis thaliana	NP_176940.1	NCBI
	Physcomitrella patens	XP_001768242.1	NCBI
	Cyanidioschyzon merolae	CMV045C	CMGP
	Gracilaria tenuistipitata	YP_063525.1	NCBI
	Chondrus crispus	YP_007627307.1	KEGG

	Cyanophora paradoxa	P48272.1	UniProtKB/Swiss-Prot
	Glaucocystis nostochinearum	-	
	Guillardia theta	NP_050684.1	NCBI
	Emiliania huxleyi	Q4G385.1	UniProtKB/Swiss-Prot
	Phaeodactylum tricornutum	YP_874435.1	NCBI
	Thalassiosira pseudonana	YP_874565.1	NCBI
	Fragilariopsis cylindrus	-	
	Ectocarpus siliculosus	YP_003289275.1	NCBI
	Aureococcus anophagefferens	-	
	Nannochloropsis gaditana	YP_007317089.1	NCBI
	Vitrella brassicaformis	Vbra_4340	EuPathDB
	Chromera velia	-	
	Bigelowiella natans	Bigna1|90258	JGI
	Paulinella chromatophora	YP_002049444.1	NCBI
	Euglena gracilis	-	
psbZ	*Prochlorococcus marinus*	WP_011129459.1	NCBI
	Synechococcus elongatus	WP_011244163.1	NCBI
	Thermosynechococcus elongatus	WP_011057802.1	NCBI
	Microcystis aeruginosa	WP_002732865.1	NCBI
	Anabaena variabilis	Q3MCF9.1	UniProtKB/Swiss-Prot
	Volvox carteri	ACY06039.1	GenBank
	Chlamydomonas reinhardtii	NP_958383.1	NCBI
	Ostreococcus tauri	YP_717234.1	NCBI
	Micromonas commoda	YP_002808636.1	NCBI
	Arabidopsis thaliana	NP_051056.1	NCBI
	Physcomitrella patens	NP_904205.1	NCBI
	Cyanidioschyzon merolae	CMV141C	CMGP
	Gracilaria tenuistipitata	YP_063609.1	NCBI
	Chondrus crispus	YP_007627390.1	KEGG
	Cyanophora paradoxa	NP_043204.1	NCBI
	Glaucocystis nostochinearum	-	
	Guillardia theta	NP_050760.1	NCBI
	Emiliania huxleyi	YP_277341.1	NCBI
	Phaeodactylum tricornutum	YP_874374.1	NCBI
	Thalassiosira pseudonana	YP_874545.1	NCBI
	Fragilariopsis cylindrus	chloroplast scaffold_95	JGI
	Ectocarpus siliculosus	-	
	Aureococcus anophagefferens	-	
	Nannochloropsis gaditana	-	
	Vitrella brassicaformis	-	
	Chromera velia	-	
	Bigelowiella natans	-	
	Paulinella chromatophora	YP_002049134.1	NCBI
	Euglena gracilis	NP_041931.1	NCBI
Psb27	*Prochlorococcus marinus*	ABM77494.1	GenBank
	Synechococcus elongatus	WP_011377542.1	NCBI
	Thermosynechococcus elongatus	NP_683253.1	NCBI
	Microcystis aeruginosa	WP_012265509.1	NCBI
	Anabaena variabilis	ABA20202.1	GenBank
	Volvox carteri	XP_002956213.1	NCBI
	Chlamydomonas reinhardtii	XP_001700736.1	NCBI
	Ostreococcus lucimarinus	XP_001419197.1	NCBI
	Micromonas commoda	XP_002508067.1	NCBI
	Arabidopsis thaliana	NP_563687.1	NCBI
	Physcomitrella patens	XP_001770919.1	NCBI
	Cyanidioschyzon merolae	CMK176C	CMGP
	Gracilaria tenuistipitata	HS981381.1	GenBank

	Chondrus crispus	-	
	Cyanophora paradoxa	Contig7118	CGP
	Glaucocystis nostochinearum	-	
	Guillardia theta	Guith1|102421	JGI
	Emiliania huxleyi	XP_005768107.1	NCBI
	Phaeodactylum tricornutum	XP_002177209.1	NCBI
	Thalassiosira pseudonana	XP_002288234.1	NCBI
	Fragilariopsis cylindrus	Fracy1|212328	JGI
	Ectocarpus siliculosus	CBN76956.1	GenBank
	Aureococcus anophagefferens	EGB06728.1	GenBank
	Nannochloropsis gaditana	EWM25384.1	GenBank
	Vitrella brassicaformis	Vbra_10350	EuPathDB
	Chromera velia	Cvel_16029	EuPathDB
	Bigelowiella natans	Bigna1|90613	JGI
	Paulinella chromatophora	YP_002049501.1	NCBI
	Euglena gracilis	ELL00006299	TBestDB
Ycf12/psb30	*Prochlorococcus marinus*	KGG13826.1	GenBank
	Synechococcus elongatus	Q0IAK1.2	UniProtKB/Swiss-Prot
	Thermosynechococcus elongatus	BAC08794.1	GenBank
	Microcystis aeruginosa	B0JM63.2	UniProtKB/Swiss-Prot
	Anabaena sp.	QBQ02713.1	GenBank
	Volvox carteri	ACY06034.1	GenBank
	Chlamydomonas reinhardtii	NP_958378.1	NCBI
	Ostreococcus tauri	YP_717256.1	NCBI
	Micromonas commoda	YP_002808610.1	NCBI
	Arabidopsis thaliana	-	
	Physcomitrella patens	NP_904215.1	NCBI
	Cyanidioschyzon merolae	CMV003C	CMGP
	Gracilaria tenuistipitata	YP_063700.1	NCBI
	Chondrus crispus	YP_007627484.1	KEGG
	Cyanophora paradoxa	AAA81244.1	GenBank
	Glaucocystis nostochinearum	-	
	Guillardia theta	NP_050717.1	NCBI
	Emiliania huxleyi	YP_277335.1	NCBI
	Phaeodactylum tricornutum	YP_874375.1	NCBI
	Thalassiosira pseudonana	YP_874544.1	NCBI
	Fragilariopsis cylindrus	chloroplast scaffold_95	JGI
	Ectocarpus siliculosus	YP_003289282.1	NCBI
	Aureococcus anophagefferens	YP_003002026.1	NCBI
	Nannochloropsis oculata	YP_008519697.1	NCBI
	Vitrella brassicaformis	-	
	Chromera velia	-	
	Bigelowiella natans	-	
	Paulinella chromatophora	YP_002049472.1	NCBI
	Euglena gracilis	CAA50084.1	GenBank
Ycf39	*Prochlorococcus marinus*	WP_011825512.1	NCBI
	Synechococcus elongatus	ABB58455.1	GenBank
	Thermosynechococcus elongatus	NP_681649.1	NCBI
	Microcystis aeruginosa	WP_002762970.1	NCBI
	Anabaena variabilis	ABA21542.1	NCBI
	Volvox carteri	XP_002958804.1	NCBI
	Chlamydomonas reinhardtii	XP_001700491.1	NCBI
	Ostreococcus lucimarinus	XP_001422150.1	NCBI
	Micromonas commoda	XP_002501159.1	NCBI
	Arabidopsis thaliana	NP_195251.1	NCBI
	Physcomitrella patens	XP_001773602.1	NCBI
	Cyanidioschyzon merolae	CMV242C	CMGP
	Gracilaria tenuistipitata	YP_063656.1	NCBI

	Chondrus crispus	YP_007627439.1	KEGG
	Cyanophora paradoxa	AAA81188.1	GenBank
	Glaucocystis nostochinearum	-	
	Guillardia theta	NP_050729.1	NCBI
	Emiliania huxleyi	YP_277427.1	NCBI
	Phaeodactylum tricornutum	YP_874364.1	NCBI
	Thalassiosira pseudonana	YP_874559.1	NCBI
	Fragilariopsis cylindrus	chloroplast scaffold_95	JGI
	Ectocarpus siliculosus	YP_003289150.1	NCBI
	Aureococcus anophagefferens	YP_003002093.1	NCBI
	Nannochloropsis gaditana	-	
	Vitrella brassicaformis	Vbra_5979	EuPathDB
	Chromera velia	Cvel_7081	EuPathDB
	Bigelowiella natans	Bigna1|92431	JGI
	Paulinella chromatophora	YP_002048699.1	NCBI
	Euglena gracilis	-	
Ycf48	*Prochlorococcus marinus*	WP_011827105.1	NCBI
	Synechococcus elongatus	WP_011377902.1	NCBI
	Thermosynechococcus elongatus	NP_682485.1	NCBI
	Microcystis aeruginosa	BAG48283.1	GenBank
	Anabaena variabilis	ABA21478.1	NCBI
	Volvox carteri	XP_002946220.1	NCBI
	Chlamydomonas reinhardtii	XP_001696194.1	NCBI
	Ostreococcus lucimarinus	XP_001420924.1	NCBI
	Micromonas commoda	XP_002502066.1	NCBI
	Arabidopsis thaliana	NP_197703.1	NCBI
	Physcomitrella patens	XP_001756806.1	NCBI
	Cyanidioschyzon merolae	CMO314C	CMGP
	Gracilaria tenuistipitata	HS979242.1	GenBank
	Chondrus crispus	XP_005713411.1	KEGG
	Cyanophora paradoxa	P48325.1	UniProtKB/Swiss-Prot
	Glaucocystis nostochinearum	-	
	Guillardia theta	XP_001713238.1	NCBI
	Emiliania huxleyi	XP_005794599.1	NCBI
	Phaeodactylum tricornutum	XP_002181754.1	NCBI
	Thalassiosira pseudonana	XP_002295048.1	NCBI
	Fragilariopsis cylindrus	Fracy1|259589	JGI
	Ectocarpus siliculosus	CBJ25908.1	GenBank
	Aureococcus anophagefferens	EGB06598.1	GenBank
	Nannochloropsis gaditana	EWM29690.1	GenBank
	Vitrella brassicaformis	Vbra_15146	EuPathDB
	Chromera velia	Cvel_32489	EuPathDB
	Bigelowiella natans	Bigna1|91760	JGI
	Paulinella chromatophora	YP_002049139.1	NCBI
	Euglena gracilis	ELL00007923	TBestDB
petA	*Prochlorococcus marinus*	WP_011825334.1	NCBI
	Synechococcus elongatus	WP_011242633.1	NCBI
	Thermosynechococcus elongatus	NP_681750.1	NCBI
	Microcystis aeruginosa	WP_012265194.1	NCBI
	Anabaena variabilis	CAB72245.1	GenBank
	Volvox carteri	ACY06009.1	GenBank
	Chlamydomonas reinhardtii	NP_958358.1	NCBI
	Ostreococcus tauri	YP_717252.1	NCBI
	Micromonas commoda	YP_002808618.1	NCBI
	Arabidopsis thaliana	NP_051072.1	NCBI
	Physcomitrella patens	NP_904189.1	NCBI
	Cyanidioschyzon merolae	CMV155C	CMGP
	Gracilaria tenuistipitata	YP_063630.1	NCBI
	Chondrus crispus	YP_007627413.1	KEGG

	Cyanophora paradoxa	NP_043244.1	NCBI
	Glaucocystis nostochinearum	-	
	Guillardia theta	NP_050751.1	NCBI
	Emiliania huxleyi	YP_277330.1	NCBI
	Phaeodactylum tricornutum	YP_874404.1	NCBI
	Thalassiosira pseudonana	YP_874531.1	NCBI
	Fragilariopsis cylindrus	chloroplast scaffold_95	JGI
	Ectocarpus siliculosus	YP_003289264.1	NCBI
	Aureococcus anophagefferens	YP_003002119.1	NCBI
	Nannochloropsis gaditana	YP_007317073.1	NCBI
	Vitrella brassicaformis	ADJ66596.1	GenBank
	Chromera velia	YP_003795261.2	NCBI
	Bigelowiella natans	YP_778563.1	NCBI
	Paulinella chromatophora	YP_002049380.1	NCBI
	Euglena gracilis	Q8GZR2.2	UniProtKB/Swiss-Prot
petB	*Prochlorococcus marinus*	ABM78920.1	GenBank
	Synechococcus elongatus	WP_011244081.1	NCBI
	Thermosynechococcus elongatus	NP_681585.1	NCBI
	Microcystis aeruginosa	WP_002798126.1	NCBI
	Anabaena variabilis	CAB72242.1	GenBank
	Volvox carteri	ACY06015.1	GenBank
	Chlamydomonas reinhardtii	NP_958365.1	NCBI
	Ostreococcus tauri	YP_717215.1	NCBI
	Micromonas commoda	YP_002808654.1	NCBI
	Arabidopsis thaliana	NP_051088.1	NCBI
	Physcomitrella patens	NP_904171.1	NCBI
	Cyanidioschyzon merolae	CMV096C	CMGP
	Gracilaria tenuistipitata	YP_063557.1	NCBI
	Chondrus crispus	YP_007627338.1	KEGG
	Cyanophora paradoxa	NP_043175.1	NCBI
	Glaucocystis nostochinearum	-	
	Guillardia theta	NP_050667.1	NCBI
	Emiliania huxleyi	YP_277309.1	NCBI
	Phaeodactylum tricornutum	YP_874393.1	NCBI
	Thalassiosira pseudonana	YP_874548.1	NCBI
	Fragilariopsis cylindrus	chloroplast scaffold_95	JGI
	Ectocarpus siliculosus	YP_003289251.1	NCBI
	Aureococcus anophagefferens	YP_003002103.1	NCBI
	Nannochloropsis gaditana	YP_007317001.1	NCBI
	Vitrella brassicaformis	ADJ66651.1	GenBank
	Chromera velia	YP_003795329.1	NCBI
	Bigelowiella natans	YP_778577.1	NCBI
	Paulinella chromatophora	YP_002049280.1	NCBI
	Euglena gracilis	NP_041942.1	NCBI
petC	*Prochlorococcus marinus*	WP_011825335.1	NCBI
	Synechococcus elongatus	WP_011242632.1	NCBI
	Thermosynechococcus elongatus	NP_681749.1	NCBI
	Microcystis aeruginosa	WP_002781457.1	NCBI
	Anabaena variabilis	ABA20011.1	NCBI
	Volvox carteri	XP_002947706.1	NCBI
	Chlamydomonas reinhardtii	XP_001698786.1	NCBI
	Ostreococcus lucimarinus	XP_001416036.1	NCBI
	Micromonas commoda	XP_002506920.1	NCBI
	Arabidopsis thaliana	NP_192237.1	NCBI
	Physcomitrella patens	XP_001758988.1	NCBI
	Cyanidioschyzon merolae	CMI281C	CMGP
	Gracilaria tenuistipitata	HS981632.1	GenBank
	Chondrus crispus	XP_005712328.1	GenBank

	Cyanophora paradoxa	Q5CC93.1	UniProtKB/Swiss-Prot
	Glaucocystis nostochinearum	-	
	Guillardia theta	XP_005821071.1	NCBI
	Emiliania huxleyi	XP_005770222.1	NCBI
	Phaeodactylum tricornutum	XP_002185824.1	NCBI
	Thalassiosira pseudonana	XP_002287488.1	NCBI
	Fragilariopsis cylindrus	GW080207.1	NCBI EST
	Ectocarpus siliculosus	CBJ29582.1	GenBank
	Aureococcus anophagefferens	XP_009038973.1	NCBI
	Nannochloropsis gaditana	EWM28243.1	GenBank
	Vitrella brassicaformis	Vbra_13677	EuPathDB
	Chromera velia	Cvel_20353	EuPathDB
	Bigelowiella natans	Q7XYM4.1	UniProtKB/Swiss-Prot
	Paulinella chromatophora	YP_002049381.1	NCBI
	Euglena gracilis	ELL00002516	TBestDB
petD	*Prochlorococcus marinus*	WP_011826790.1	NCBI
	Synechococcus elongatus	AAA98849.1	GenBank
	Thermosynechococcus elongatus	NP_681586.1	NCBI
	Microcystis aeruginosa	WP_002738373.1	NCBI
	Anabaena variabilis	ABA23049.1	NCBI
	Volvox carteri	ACY06008.1	GenBank
	Chlamydomonas reinhardtii	NP_958359.1	NCBI
	Ostreococcus tauri	XP_003080162.1	NCBI
	Micromonas commoda	XP_002499519.1	NCBI
	Arabidopsis thaliana	NP_051089.1	NCBI
	Physcomitrella patens	NP_904170.1	NCBI
	Cyanidioschyzon merolae	CMV097C	CMGP
	Gracilaria tenuistipitata	YP_063556.1	NCBI
	Chondrus crispus	YP_007627339.1	KEGG
	Cyanophora paradoxa	NP_043174.1	NCBI
	Glaucocystis nostochinearum	-	
	Guillardia theta	NP_050666.1	NCBI
	Emiliania huxleyi	YP_277310.1	NCBI
	Phaeodactylum tricornutum	YP_874392.1	NCBI
	Thalassiosira pseudonana	YP_874549.1	NCBI
	Fragilariopsis cylindrus	chloroplast scaffold_95	JGI
	Ectocarpus siliculosus	YP_003289252.1	NCBI
	Aureococcus anophagefferens	YP_003002102.1	NCBI
	Nannochloropsis salina	YP_008519754.1	NCBI
	Vitrella brassicaformis	ADJ66594.1	NCBI
	Chromera velia	YP_003795262.1	NCBI
	Bigelowiella natans	YP_778578.1	NCBI
	Paulinella chromatophora	YP_002049281.1	NCBI
	Euglena gracilis	Q84TU6.1	UniProtKB/Swiss-Prot
petG	*Prochlorococcus marinus*	NP_875534.1	NCBI
	Synechococcus elongatus	WP_011378050.1	NCBI
	Thermosynechococcus elongatus	NP_681667.1	NCBI
	Microcystis aeruginosa	WP_002738052.1	NCBI
	Anabaena variabilis	Q3M5W2.1	UniProtKB/Swiss-Prot
	Volvox carteri	ACY06042.1	GenBank
	Chlamydomonas reinhardtii	ACJ50132.1	GenBank
	Ostreococcus tauri	YP_717263.1	NCBI
	Micromonas commoda	YP_002808617.1	NCBI
	Arabidopsis thaliana	NP_051078.1	NCBI
	Physcomitrella patens	NP_904181.1	NCBI
	Cyanidioschyzon merolae	CMV139C	CMGP

	Gracilaria tenuistipitata	YP_063611.1	NCBI
	Chondrus crispus	YP_007627392.1	KEGG
	Cyanophora paradoxa	NP_043220.1	NCBI
	Glaucocystis nostochinearum	-	
	Guillardia theta	NP_050762.1	NCBI
	Emiliania huxleyi	YP_277389.1	NCBI
	Phaeodactylum tricornutum	YP_874380.1	NCBI
	Thalassiosira pseudonana	YP_874539.1	NCBI
	Fragilariopsis cylindrus	chloroplast scaffold_95	JGI
	Ectocarpus siliculosus	YP_003289212.1	NCBI
	Aureococcus anophagefferens	YP_003002089.1	NCBI
	Nannochloropsis gaditana	YP_007316993.1	NCBI
	Vitrella brassicaformis	ADJ66580.1	GenBank
	Chromera velia	YP_003795328.1	NCBI
	Bigelowiella natans	YP_778564.1	NCBI
	Paulinella chromatophora	YP_002049395.1	NCBI
	Euglena gracilis	NP_041905.1	NCBI
petL	*Prochlorococcus marinus*	WP_011129552.1	NCBI
	Synechococcus elongatus	WP_011243703.1	NCBI
	Thermosynechococcus elongatus	YP_654185.1	NCBI
	Microcystis aeruginosa	CCI38222.1	GenBank
	Anabaena variabilis	Q3M4V0.1	UniProtKB/Swiss-Prot
	Volvox carteri	ACY06005.1	GenBank
	Chlamydomonas reinhardtii	NP_958424.2	NCBI
	Ostreococcus tauri	CEG00971.1	GenBank
	Micromonas commoda	-	
	Arabidopsis thaliana	P56776.2	UniProtKB/Swiss-Prot
	Physcomitrella patens	NP_904182.1	NCBI
	Cyanidioschyzon merolae	CMV235C	CMGP
	Gracilaria tenuistipitata	-	
	Chondrus crispus	CHC_920	KEGG
	Cyanophora paradoxa	P48102.1	UniProtKB/Swiss-Prot
	Glaucocystis nostochinearum	-	
	Guillardia theta	NP_050725.1	NCBI
	Emiliania huxleyi	YP_277357.1	NCBI
	Phaeodactylum tricornutum	YP_874367.1	NCBI
	Thalassiosira pseudonana	YP_874556.1	NCBI
	Fragilariopsis cylindrus	chloroplast scaffold_95	JGI
	Ectocarpus siliculosus	YP_003289278.1	NCBI
	Aureococcus anophagefferens	YP_003002021.1	NCBI
	Nannochloropsis salina	AHX25514.1	GenBank
	Vitrella brassicaformis	-	
	Chromera velia	-	
	Bigelowiella natans	-	
	Paulinella chromatophora	-	
	Euglena gracilis	-	
petM	*Prochlorococcus marinus*	ABM77601.1	GenBank
	Synechococcus elongatus	WP_011243990.1	NCBI
	Thermosynechococcus elongatus	Q8DJ15.1	UniProtKB/Swiss-Prot
	Microcystis aeruginosa	WP_012265655.1	NCBI
	Anabaena variabilis	CAC12858.1	GenBank
	Volvox carteri	XP_002953725.1	NCBI
	Chlamydomonas reinhardtii	XP_001694096.1	NCBI
	Ostreococcus lucimarinus	XP_001417333.1	NCBI
	Micromonas commoda	XP_002505123.1	NCBI
	Arabidopsis thaliana	CAA06667.1	GenBank

	Physcomitrella patens	XP_001754688.1	NCBI
	Cyanidioschyzon merolae	CMV147C	CMGP
	Gracilaria tenuistipitata	-	
	Chondrus crispus	CHC_680	KEGG
	Cyanophora paradoxa	P48366.1	UniProtKB/Swiss-Prot
	Glaucocystis nostochinearum	-	
	Guillardia theta	NP_050756.1	NCBI
	Emiliania huxleyi	Q4G3B3.1	UniProtKB/Swiss-Prot
	Phaeodactylum tricornutum	YP_874382.1	NCBI
	Thalassiosira pseudonana	YP_874538.1	NCBI
	Fragilariopsis cylindrus	chloroplast scaffold_95	JGI
	Ectocarpus siliculosus	YP_003289217.1	NCBI
	Aureococcus anophagefferens	YP_003002121.1	NCBI
	Nannochloropsis gaditana	AHX25099.1	GenBank
	Vitrella brassicaformis	-	
	Chromera velia	-	
	Bigelowiella natans	-	
	Paulinella chromatophora	-	
	Euglena gracilis	-	
petN	*Prochlorococcus marinus*	A2C9Y5.1	UniProtKB/Swiss-Prot
	Synechococcus elongatus	ABB56507.1	GenBank
	Thermosynechococcus elongatus	NP_681617.1	NCBI
	Microcystis aeruginosa	B0JID4.1	UniProtKB/Swiss-Prot
	Anabaena variabilis	P61049.1	UniProtKB/Swiss-Prot
	Volvox carteri	XP_002952299.1	NCBI
	Chlamydomonas reinhardtii	XP_001698251.1	NCBI
	Ostreococcus lucimarinus	XP_001420753.1	NCBI
	Micromonas commoda	XP_002503160.1	NCBI
	Arabidopsis thaliana	NP_051052.1	NCBI
	Physcomitrella patens	NP_904169.1	NCBI
	Cyanidioschyzon merolae	CMV148C	CMGP
	Gracilaria tenuistipitata	YP_063620.1	NCBI
	Chondrus crispus	CHC_675	KEGG
	Cyanophora paradoxa	NP_043216.1	NCBI
	Glaucocystis nostochinearum	-	
	Guillardia theta	NP_050755.1	NCBI
	Emiliania huxleyi	YP_277355.1	NCBI
	Phaeodactylum tricornutum	YP_874383.1	NCBI
	Thalassiosira pseudonana	YP_874537.1	NCBI
	Fragilariopsis cylindrus	chloroplast scaffold_95	
	Ectocarpus siliculosus	YP_003289216.1	NCBI
	Aureococcus anophagefferens	YP_003002122.1	NCBI
	Nannochloropsis gaditana	YP_007317062.1	NCBI
	Vitrella brassicaformis	ADJ66604.1	GenBank
	Chromera velia	-	
	Bigelowiella natans	Bigna1|87265	JGI
	Paulinella chromatophora	B1X3V8.1	UniProtKB/Swiss-Prot
	Euglena gracilis	EGL00000097	TBestDB
atpA	*Prochlorococcus marinus*	WP_011825158.1	NCBI
	Synechococcus elongatus	WP_011377539.1	NCBI
	Thermosynechococcus elongatus	NP_681225.1	NCBI
	Microcystis aeruginosa	WP_012267463.1	NCBI
	Anabaena variabilis	WP_011319378.1	NCBI
	Volvox carteri	ACY06056.1	GenBank

	Chlamydomonas reinhardtii	NP_958406.1	NCBI
	Ostreococcus tauri	YP_717250.1	NCBI
	Micromonas commoda	YP_002808620.1	NCBI
	Arabidopsis thaliana	NP_051044.1	NCBI
	Physcomitrella patens	NP_904216.1	NCBI
	Cyanidioschyzon merolae	CMV225C	CMGP
	Gracilaria tenuistipitata	YP_063652.1	NCBI
	Chondrus crispus	YP_007627435.1	NCBI
	Cyanophora paradoxa	NP_043222.1	NCBI
	Glaucocystis nostochinearum	-	
	Guillardia theta	NP_050732.1	NCBI
	Emiliania huxleyi	YP_277370.1	NCBI
	Phaeodactylum tricornutum	YP_874426.1	NCBI
	Thalassiosira pseudonana	YP_874506.1	NCBI
	Fragilariopsis cylindrus	chloroplast scaffold_95	JGI
	Ectocarpus siliculosus	YP_003289250.1	NCBI
	Aureococcus anophagefferens	YP_003002038.1	NCBI
	Nannochloropsis gaditana	YP_007317088.1	NCBI
	Vitrella brassicaformis	ADJ66583.1	GenBank
	Chromera velia	YP_003795331.1	NCBI
	Bigelowiella natans	YP_778559.1	NCBI
	Paulinella chromatophora	YP_002048865.1	NCBI
	Euglena gracilis	NP_041928.1	NCBI
atpB	*Prochlorococcus marinus*	ABM77254.1	GenBank
	Synechococcus elongatus	WP_011244097.1	NCBI
	Thermosynechococcus elongatus	NP_681315.1	NCBI
	Microcystis aeruginosa	WP_012263845.1	NCBI
	Anabaena variabilis	ABA21916.1	NCBI
	Volvox carteri	ACY05995.1	GenBank
	Chlamydomonas reinhardtii	NP_958414.1	NCBI
	Ostreococcus tauri	YP_717213.1	NCBI
	Micromonas commoda (mt)	XP_002507229.1	NCBI
	Arabidopsis thaliana	NP_051066.1	NCBI
	Physcomitrella patens	NP_904195.1	NCBI
	Cyanidioschyzon merolae	CMV196C	CMGP
	Gracilaria tenuistipitata	YP_063636.1	NCBI
	Chondrus crispus	YP_007627419.1	KEGG
	Cyanophora paradoxa	NP_043241.1	NCBI
	Glaucocystis nostochinearum	-	
	Guillardia theta	NP_050748.1	NCBI
	Emiliania huxleyi	YP_277339.1	NCBI
	Phaeodactylum tricornutum	YP_874407.1	NCBI
	Thalassiosira pseudonana	YP_874528.1	NCBI
	Fragilariopsis cylindrus (mt)	Fracy1|267742	JGI
	Ectocarpus siliculosus	YP_003289267.1	NCBI
	Aureococcus anophagefferens	YP_003002116.1	NCBI
	Nannochloropsis gaditana	YP_007317076.1	NCBI
	Vitrella brassicaformis	ADJ66641.1	GenBank
	Chromera velia	YP_003795295.2/ YP_003795258.2	NCBI
	Bigelowiella natans	YP_778598.1	NCBI
	Paulinella chromatophora	YP_002048874.1	NCBI
	Euglena gracilis	NP_041941.1	NCBI
atpC	*Prochlorococcus marinus*	WP_011825159.1	NCBI
	Synechococcus elongatus	WP_011377540.1	NCBI
	Thermosynechococcus elongatus	NP_681175.1	NCBI
	Microcystis aeruginosa	WP_002800073.1	NCBI
	Anabaena variabilis	WP_011319377.1	NCBI
	Volvox carteri	XP_002945818.1	NCBI
	Chlamydomonas reinhardtii	XP_001696335.1	NCBI

	Ostreococcus tauri	XP_003081102.1	NCBI
	Micromonas commoda	XP_002501219.1	NCBI
	Arabidopsis thaliana	NP_567265.1	NCBI
	Physcomitrella patens	XP_001783653.1	NCBI
	Cyanidioschyzon merolae	CMQ087C	CMGP
	Gracilaria tenuistipitata	-	
	Chondrus crispus	CO652269.1	GenBank
	Cyanophora paradoxa	Contig7165	CGP
	Glaucocystis nostochinearum	-	
	Guillardia theta	EKX51948.1	GenBank
	Emiliania huxleyi	XP_005761783.1	NCBI
	Phaeodactylum tricornutum	AAO43198.1	GenBank
	Thalassiosira pseudonana	XP_002289138.1	NCBI
	Fragilariopsis cylindrus	Fracy1|208175	JGI
	Ectocarpus siliculosus	CBN76659.1	GenBank
	Aureococcus anophagefferens	EGB12392.1	GenBank
	Nannochloropsis gaditana (mt)	NGA_0035102	KEGG
	Vitrella brassicaformis	Vbra_19020	EuPathDB
	Chromera velia	Cvel_189	EuPathDB
	Bigelowiella natans	AAP79136.1	GenBank
	Paulinella chromatophora	YP_002048866.1	NCBI
	Euglena gracilis	-	
atpD	*Prochlorococcus marinus*	WP_011130836.1	NCBI
	Synechococcus elongatus	WP_011243490.1	NCBI
	Thermosynechococcus elongatus	NP_681224.1	NCBI
	Microcystis aeruginosa	WP_002763366.1	NCBI
	Anabaena variabilis	WP_011319379.1	NCBI
	Volvox carteri	XP_002957070.1	NCBI
	Chlamydomonas reinhardtii	XP_001701377.1	NCBI
	Ostreococcus lucimarinus	XP_001418641.1	NCBI
	Micromonas commoda	XP_002499540.1	NCBI
	Arabidopsis thaliana	NP_192703.1	NCBI
	Physcomitrella patens	XP_001771366.1	NCBI
	Cyanidioschyzon merolae	CMV224C	CMGP
	Gracilaria tenuistipitata	YP_063651.1	NCBI
	Chondrus crispus	XP_005710034.1	NCBI
	Cyanophora paradoxa	-	
	Glaucocystis nostochinearum	-	
	Guillardia theta	NP_050733.1	NCBI
	Emiliania huxleyi	YP_277369.1	NCBI
	Phaeodactylum tricornutum	YP_874425.1	NCBI
	Thalassiosira pseudonana	YP_874505.1	NCBI
	Fragilariopsis cylindrus	Fracy1|260675	JGI
	Ectocarpus siliculosus	YP_003289249.1	NCBI
	Aureococcus anophagefferens	YP_003002037.1	NCBI
	Nannochloropsis gaditana	NGA_0240301	KEGG
	Vitrella brassicaformis (mt)	Vbra_17642	EuPathDB
	Chromera velia (mt)	Cvel_20402	EuPathDB
	Bigelowiella natans	AAP79166.1	GenBank
	Paulinella chromatophora	YP_002048864.1	NCBI
	Euglena gracilis	ELL00002012	TBestDB
atpE	*Prochlorococcus marinus*	ABM77253.1	GenBank
	Synechococcus elongatus	WP_011244096.1	NCBI
	Thermosynechococcus elongatus	NP_681316.1	NCBI
	Microcystis aeruginosa	WP_002735380.1	NCBI
	Anabaena variabilis	ABA21915.1	NCBI
	Volvox carteri	XP_002954956.1	NCBI
	Chlamydomonas reinhardtii	NP_958379.1	NCBI
	Ostreococcus tauri	YP_717212.1	NCBI
	Micromonas commoda	YP_002808656.1	NCBI

	Arabidopsis thaliana	NP_051065.1	NCBI
	Physcomitrella patens	NP_904196.1	NCBI
	Cyanidioschyzon merolae	CMV195C	CMGP
	Gracilaria tenuistipitata	YP_063635.1	NCBI
	Chondrus crispus	CHC_760	KEGG
	Cyanophora paradoxa	NP_043242.1	NCBI
	Glaucocystis nostochinearum	-	
	Guillardia theta	NP_050749.1	NCBI
	Emiliania huxleyi	YP_277338.1	NCBI
	Phaeodactylum tricornutum	YP_874406.1	NCBI
	Thalassiosira pseudonana	YP_874529.1	NCBI
	Fragilariopsis cylindrus	-	
	Ectocarpus siliculosus	YP_003289266.1	NCBI
	Aureococcus anophagefferens	YP_003002117.1	NCBI
	Nannochloropsis gaditana	YP_007317075.1	NCBI
	Vitrella brassicaformis	-	
	Chromera velia	-	
	Bigelowiella natans	YP_778599.1	NCBI
	Paulinella chromatophora	YP_002048873.1	NCBI
	Euglena gracilis	NP_041940.1	NCBI
atpF	*Prochlorococcus marinus*	WP_011825157.1	NCBI
	Synechococcus elongatus	WP_011377538.1	NCBI
	Thermosynechococcus elongatus	WP_011056288.1	NCBI
	Microcystis aeruginosa	WP_002795846.1	NCBI
	Anabaena variabilis	ABA22228.1	NCBI
	Volvox carteri	ACY06062.1	GenBank
	Chlamydomonas reinhardtii	NP_958410.1	NCBI
	Ostreococcus tauri	YP_717249.1	NCBI
	Micromonas commoda	YP_002808621.1	NCBI
	Arabidopsis thaliana	NP_051045.1	NCBI
	Physcomitrella patens	NP_904217.1	NCBI
	Cyanidioschyzon merolae	CMV223C	CMGP
	Gracilaria tenuistipitata	YP_063650.1	NCBI
	Chondrus crispus	CHC_835	KEGG
	Cyanophora paradoxa	NP_043224.1	NCBI
	Glaucocystis nostochinearum	-	
	Guillardia theta	NP_050734.1	NCBI
	Emiliania huxleyi	YP_277368.1	NCBI
	Phaeodactylum tricornutum	YP_874424.1	NCBI
	Thalassiosira pseudonana	YP_874504.1	NCBI
	Fragilariopsis cylindrus	chloroplast scaffold_95	JGI
	Ectocarpus siliculosus	YP_003289248.1	NCBI
	Aureococcus anophagefferens	YP_003002036.1	NCBI
	Nannochloropsis gaditana	YP_007317087.1	NCBI
	Vitrella brassicaformis	-	
	Chromera velia	-	
	Bigelowiella natans	YP_778558.1	NCBI
	Paulinella chromatophora	YP_002048863.1	NCBI
	Euglena gracilis	NP_041927.1	NCBI
atpG	*Prochlorococcus marinus*	WP_011825156.1	NCBI
	Synechococcus elongatus	WP_011243492.1	NCBI
	Thermosynechococcus elongatus	NP_681222.1	NCBI
	Microcystis aeruginosa	WP_002795845.1	NCBI
	Anabaena variabilis	WP_011319381.1	NCBI
	Volvox carteri	XP_002957122.1	NCBI
	Chlamydomonas reinhardtii	XP_001697332.1	NCBI
	Ostreococcus tauri	XP_003074143.1	NCBI
	Micromonas commoda	XP_002507222.1	NCBI
	Arabidopsis thaliana	NP_194953.1	NCBI
	Physcomitrella patens	XP_001754735.1	NCBI

	Cyanidioschyzon merolae	CMV222C	CMGP
	Gracilaria tenuistipitata	YP_063649.1	NCBI
	Chondrus crispus	CHC_830	KEGG
	Cyanophora paradoxa	NP_043225.1	NCBI
	Glaucocystis nostochinearum	-	
	Guillardia theta	NP_050735.1	NCBI
	Emiliania huxleyi	YP_277367.1	NCBI
	Phaeodactylum tricornutum	YP_874423.1	NCBI
	Thalassiosira pseudonana	XP_002297549.1	NCBI
	Fragilariopsis cylindrus	chloroplast scaffold_95	JGI
	Ectocarpus siliculosus	YP_003289247.1	NCBI
	Aureococcus anophagefferens	YP_003002035.1	NCBI
	Nannochloropsis gaditana	AGI98678.1	GenBank
	Vitrella brassicaformis	-	
	Chromera velia	-	
	Bigelowiella natans	Bigna1|47572	JGI
	Paulinella chromatophora	YP_002048862.1	NCBI
	Euglena gracilis	EGL00002701	TBestDB
atpH	*Prochlorococcus marinus*	ABM77231.1	GenBank
	Synechococcus elongatus	WP_011243493.1	NCBI
	Thermosynechococcus elongatus	NP_681221.1	NCBI
	Microcystis aeruginosa	WP_002740068.1	NCBI
	Anabaena variabilis	WP_010994186.1	NCBI
	Volvox carteri	ACY06061.1	GenBank
	Chlamydomonas reinhardtii	NP_958409.1	NCBI
	Ostreococcus tauri	YP_717248.1	NCBI
	Micromonas commoda	YP_002808622.1	NCBI
	Arabidopsis thaliana	NP_051046.1	NCBI
	Physcomitrella patens	NP_904218.1	NCBI
	Cyanidioschyzon merolae	CMV221C	CMGP
	Gracilaria tenuistipitata	YP_063648.1	NCBI
	Chondrus crispus	CHC_825	KEGG
	Cyanophora paradoxa	NP_043226.1	NCBI
	Glaucocystis nostochinearum	-	
	Guillardia theta	NP_050736.1	NCBI
	Emiliania huxleyi	YP_277366.1	NCBI
	Phaeodactylum tricornutum	YP_874422.1	NCBI
	Thalassiosira pseudonana	YP_874502.1	NCBI
	Fragilariopsis cylindrus	chloroplast scaffold_95	JGI
	Ectocarpus siliculosus	YP_003289246.1	NCBI
	Aureococcus anophagefferens	YP_003002034.1	NCBI
	Nannochloropsis gaditana	YP_007317086.1	NCBI
	Vitrella brassicaformis	ADJ66585.1	GenBank
	Chromera velia	YP_003795298.1	NCBI
	Bigelowiella natans	YP_778557.1	NCBI
	Paulinella chromatophora	YP_002048861.1	NCBI
	Euglena gracilis	NP_041926.1	NCBI
atpI	*Prochlorococcus marinus*	WP_011825154.1	NCBI
	Synechococcus elongatus	BAD79372.1	GenBank
	Thermosynechococcus elongatus	NP_681220.1	NCBI
	Microcystis aeruginosa	WP_002739857.1	NCBI
	Anabaena variabilis	WP_011319382.1	NCBI
	Volvox carteri	ACY05999.1	GenBank
	Chlamydomonas reinhardtii	ACJ50149.1	GenBank
	Ostreococcus tauri	YP_717247.1	NCBI
	Micromonas commoda	YP_002808623.1	NCBI
	Arabidopsis thaliana	BAA84373.1	GenBank
	Physcomitrella patens	NP_904219.1	NCBI
	Cyanidioschyzon merolae	CMV220C	CMGP
	Gracilaria tenuistipitata	YP_063647.1	NCBI

Chondrus crispus	CHC_820	KEGG
Cyanophora paradoxa	NP_043226.1	NCBI
Glaucocystis nostochinearum	-	
Guillardia theta	NP_050737.1	NCBI
Emiliania huxleyi	YP_277365.2	NCBI
Phaeodactylum tricornutum	YP_874421.1	NCBI
Thalassiosira pseudonana	YP_874501.1	NCBI
Fragilariopsis cylindrus	chloroplast scaffold_95	JGI
Ectocarpus siliculosus	YP_003289245.1	NCBI
Aureococcus anophagefferens	YP_003002033.1	NCBI
Nannochloropsis gaditana	YP_007317086.1	NCBI
Vitrella brassicaformis	ADJ66586.1	GenBank
Chromera velia	YP_003795310.1	NCBI
Bigelowiella natans	YP_778556.1	NCBI
Paulinella chromatophora	YP_002048860.1	NCBI
Euglena gracilis	NP_041925	NCBI

Supplementary Table S2: Manganese and iron superoxide dismutases and accession numbers.

Taxon	Accession Number	Database
Anabaena cylindrica	AFZ55773.1<br>AFZ58548.1	NCBI/GenBank
Arabidopsis thaliana	NP_187703.1<br>NP_191194.1<br>NP_197722.1<br>NP_199923.1<br>NP_849441.1<br>NP_001030670.1<br>NP_001031710.1	NCBI/GenBank
Archaeon I	AJF59898.1<br>AJF61931.1<br>KHO46305.1<br>KHO55439.1	NCBI/GenBank
Aureococcus anophagefferens	XP_009037702.1<br>XP_009038137.1	NCBI/GenBank
Babesia bovis	XP_001611068.1	NCBI/GenBank
Bigelowiella natans	Bigna1|24544<br>Bigna1|41283<br>Bigna1|141114	JGI
Caenorhabditis elegans	NP_492290.1<br>NP_510764.1	NCBI/GenBank
Chlamydomonas reinhardtii	XP_001690591.1<br>XP_001690936.1<br>XP_001691748.1<br>XP_001695947.1<br>XP_001699077.1<br>XP_001700058.1	NCBI/GenBank
Chondrus crispus	XP_005714949.1	

	XP_005716849.1	
Chromera velia	Cvel_3019<br>Cvel 4244<br>Cvel_7136<br>Cvel_26697	EuPathdb
Coxiella burnetii	3TQJ|A<br>AIT62637.1<br>WP_017252695.1<br>WP_040070417.1<br>WP_040953557.1<br>WP_042527212.1	NCBI/GenBank
Crypthecodinium cohnii	CAMPEP_0193864674	iMicrobe[a]
Cryptosporidium parvum	AAQ09593.1<br>XP_001388283.1	NCBI/GenBank
Cyanidioschyzon merolae	CMN023C<br>CMR158C<br>CMT028C	CMGP
Drosophila melanogaster	NP_001286503.1	
Ectocarpus siliculosus	CBJ28064.1<br>CBJ30867.1<br>CBJ48355.1<br>CBN76241.1<br>CBN79353.1	NCBI/GenBank
Eimeria tenella	XP_013232031.1<br>XP_013232304.1<br>XP_013234977.1	NCBI/GenBank
Emiliania huxleyi	EOD31646.1	NCBI/GenBank
Encephalitozoon cuniculi	CAD26018.2	NCBI/GenBank
Escherichia coli	NP_416173.1<br>NP_418344.3	NCBI/GenBank
Eutreptiella gymnastica-like	CAMPEP_0174321430<br>CAMPEP_0174338960	iMicrobe
Gregarina niphandrodes	XP_011129742.1	NCBI/GenBank
Guillardia theta	XP_005819012.1<br>XP_005829864.1<br>XP_005832985.1<br>XP_005841089.1<br>XP_005841396.1	NCBI/GenBank
Gymnodinium sp.	CAMPEP_0117456956<br>CAMPEP_0117464096<br>CAMPEP_0117475122<br>CAMPEP_0117556838<br>CAMPEP_0117582460	iMicrobe
Homo sapiens	NP_001019636.1<br>NP_001019637.1	NCBI/GenBank
Idiomarina sp.	WP_053952566.1	NCBI/GenBank
Karenia brevis	CAMPEP_0188874120<br>CAMPEP_0188886508<br>CAMPEP_0188906418<br>CAMPEP_0188963926<br>CAMPEP_0188978854<br>CAMPEP_0188984554<br>CAMPEP_0188992448<br>CAMPEP_0189001786	iMicrobe

Lingulodinium polyedrum	CAMPEP_0190134060	iMicrobe
Marine Group I	WP_048069774.1<br>WP_048079200.1	NCBI/GenBank
Marinobacter sp.	WP_008171376.1<br>WP_011785497.1<br>WP_014870267.1<br>WP_036210113.1	NCBI/GenBank
Methanobacterium	WP_048081171.1	NCBI/GenBank
Methanosarcina sp.	WP_048128602.1<br>WP_048137433.1	NCBI/GenBank
Microcystis aeruginosa	BAG05221.1<br>WP_045358983.1<br>WP_046662956.1	NCBI/GenBank
Micromonas sp.	XP_003058055.1	NCBI/GenBank
Nannochloropsis gaditana	EWM21824.1<br>EWM22517.1	NCBI/GenBank
Nitrosopumilus sp.	WP_014962683.1<br>WP_014964564.1<br>WP_014964930.1	
Oxyrrhis marina	CAMPEP_0190312732<br>CAMPEP_0190354292<br>CAMPEP_0190383648<br>PEP_0190378606	iMicrobe
Paramecium tetraurelia	XP_001433955.1<br>XP_001461921.1	NCBI/GenBank
Paulinella chromatophora	ACB42592.1	NCBI/GenBank
Penicillium digitatum	EKV07912.1<br>EKV14618.1	NCBI/GenBank
Perkinsus marinus	XP_002765946.1<br>XP_002773398.1<br>XP_002775938.1<br>XP_002776371.1<br>XP_002776372.1<br>XP_002784134.1<br>XP_002788749.1<br>XP_002788750.1	NCBI/GenBank
Persicobacter sp.	WP_060685238.1<br>WP_060687327.1	
Phaeodactylum tricornutum	XP_002177253.1<br>XP_002180497.1	NCBI/GenBank
Physcomitrella patens	XP_001751975.1<br>XP_001756362.1<br>XP_001774291.1	NCBI/GenBank
Phytophthora infestans	XP_002905014.1<br>XP_002905257.1	
Plasmodiophora brassicae	CEO96504.1<br>CEP02381.1	NCBI/GenBank
Plasmodium falciparum	KOB59380.1<br>KOB60722.1	NCBI/GenBank
Pyropia haitanensis	AFS17267.1	NCBI/GenBank
Rheinheimera	GAB59341.1	NCBI/GenBank
Saccharomyces cerevisiae	NP_011872.1	NCBI/GenBank
Solanum tuberosum	AAO16563.1<br>XP_006350369.1	NCBI/GenBank

	XP_006358755.1<br>XP_015168847.1	
Symbiodinium sp.	AAX99422.1	NCBI/GenBank
Synechococcus elongatus	BAD78927.1	NCBI/GenBank
Tetrahymena thermophila	XP_001010506.1	NCBI/GenBank
Thalassiosira pseudonana	XP_002288307.1<br>XP_002290645.1<br>XP_002295691.1	NCBI/GenBank
Theileria parva	XP_763660.1<br>XP_766677.1	NCBI/GenBank
Toxoplasma gondii	XP_002364786.1<br>XP_002364798.1<br>XP_002364800.1	NCBI/GenBank
Trichomonas vaginalis	XP_001276944.1<br>XP_001305233.1<br>XP_001307775.1<br>XP_001317169.1<br>XP_001320475.1<br>XP_001320923.1<br>XP_001324547.1	NCBI/GenBank
Trypanosoma brucei	XP_829648.1<br>XP_829560.1<br>XP_829639.1<br>XP_845007.1<br>XP_845521.1	NCBI/GenBank
Vitrella brassicaformis	Vbra_1709<br>Vbra_9665<br>Vbra_9701<br>Vbra_10461<br>Vbra_13639<br>Vbra_19404<br>Vbra_23058	EuPathdb
Volvox carteri	XP_002946030.1<br>XP_002946031.1<br>XP_002946032.1<br>XP_002946659.1<br>XP_002951375.1<br>XP_002958078.1<br>XP_002958467.1	NCBI/GenBank
Xanthomonas oryzae	AAW75966.1<br>ACD58439.1<br>ACD60067.1<br>AKK64553.1<br>AKN93588.1<br>AKO08548.1<br>ALS94977.1<br>ALS95224.1<br>BAE69796.1<br>WP_019299923.1<br>WP_019302872.1<br>WP_019305192.1	NCBI/GenBank
Xenorhabdus bovienii	WP_012986675.1<br>WP_012988922.1<br>WP_038183765.1	NCBI/GenBank

WP_038190998.1<br>WP_038197542.1<br>WP_038197721.1<br>WP_038221335.1<br>WP_038224240.1<br>WP_038242940.1<br>WP_038248008.1<br>WP_038254067.1<br>WP_038255860.1

[a] http://imicrobe.us/

Supplementary Table S3: SignalP and TransitP results for superoxide dismutases in *Chromera velia* and *Vitrella brassicaformis*. For sequences where a signal peptide is present the length of the signal peptide sequence is indicated. For each sequence with a positive result, the presence and length of an additional targeting peptide sequence is given. Bolded accession numbers indicate proteins that were identified as PSI subunits in 2D PAGE analyses.

Taxon	Accession number	Predicted function	Signal P length	TargetP *
Chromera velia	**Cvel_3019**	FeSOD	17 aa	24
	Cvel_4244	MnSOD	-	-
	**Cvel_7136**	FeSOD	19 aa	83
	Cvel_26697	MnSOD	-	-
Vitrella brassicaformis	Vbra_1709	MnSOD	-	-
	Vbra_9665	FeSOD	-	-
	Vbra_9701	MnSOD	18 aa	-
	**Vbra_10461**	FeSOD	18 aa	14
	Vbra_13639	FeSOD	16 aa	25
	Vbra_19404	MnSOD	19 aa	-
	Vbra_23058	FeSOD	-	-

*for peptide sequence after signal peptide removal

Supplementary Table S4: Full HECTAR (optimized for heterokont proteins) results for superoxide dismutase sequences from *Chromera velia* and *Vitrella brassicaformis*. Bolded accession numbers indicate proteins that were identified as PSI subunits in 2D PAGE analyses.

Taxon	sequence	Predicted targeting category	Signal peptide score	Type ii signal anchor score	Chloroplast score	Mitochondrion score	Other score
Chromera velia	**Cvel_7136**	Signal peptide	0.6791	0.0556	0.3803	-	-
	**Cvel_3019**	chloroplast	0.6375	0.0611	0.7929	-	-
	Cvel_4244	Signal peptide	0.6403	0.0624	0.0485	-	-
	Cvel_26697	Other localisation	0.0694	0.0248	-	0.1176	0.8824
Vitrella brassicaformis	Vbra_19404	Signal peptide	0.7242	0.0552	0.0188	-	-
	Vbra_9665	Other localisation	0.1008	0.0309	-	0.1033	0.8967
	Vbra_23058	mitochondrion	0.1221	0.0341	-	0.6766	0.3234
	Vbra_1709	Other localisation	0.0843	0.0475	-	0.1080	0.892
	Vbra_13639	chloroplast	0.7792	0.0549	0.7402	-	-
	**Vbra_10461**	chloroplast	0.7518	0.0578	0.9238	-	-
	Vbra_9701	Signal peptide	0.8132	0.0522	0.0400	-	-

Supplementary Table S5: HECTAR^SEC (all eukaryotes) results for superoxide dismutase sequences from from *Chromera velia* and *Vitrella brassicaformis*. Bolded accession numbers indicate proteins that were identified as PSI subunits in 2D PAGE analyses.

Taxon	sequence	Predicted targeting category	Signal peptide score	Type ii signal anchor score	Other score
Chromera velia	**Cvel_7136**	Signal peptide	0.6791	0.0556	0.2653
	**Cvel_3019**	Signal peptide	0.6376	0.0611	0.3013
	Cvel_4244	Signal peptide	0.6403	0.0624	0.2973
	Cvel_26697	No signal peptide or anchor	0.0694	0.0248	0.9058
Vitrella brassicaformis	Vbra_19404	Signal peptide	0.7242	0.0552	0.2206
	Vbra_9665	No signal peptide or anchor	0.1008	0.0309	0.8683
	Vbra_23058	No signal peptide or anchor	0.1221	0.0341	0.8438
	Vbra_1709	No sigal peptide or anchor	0.0843	0.0275	0.8882
	Vbra_13639	Signal peptide	0.7792	0.0549	0.1659
	**Vbra_10461**	Signal peptide	0.7518	0.0578	0.1904

	Vbra_9701	Signal peptide	0.8132	0.0522	0.1346

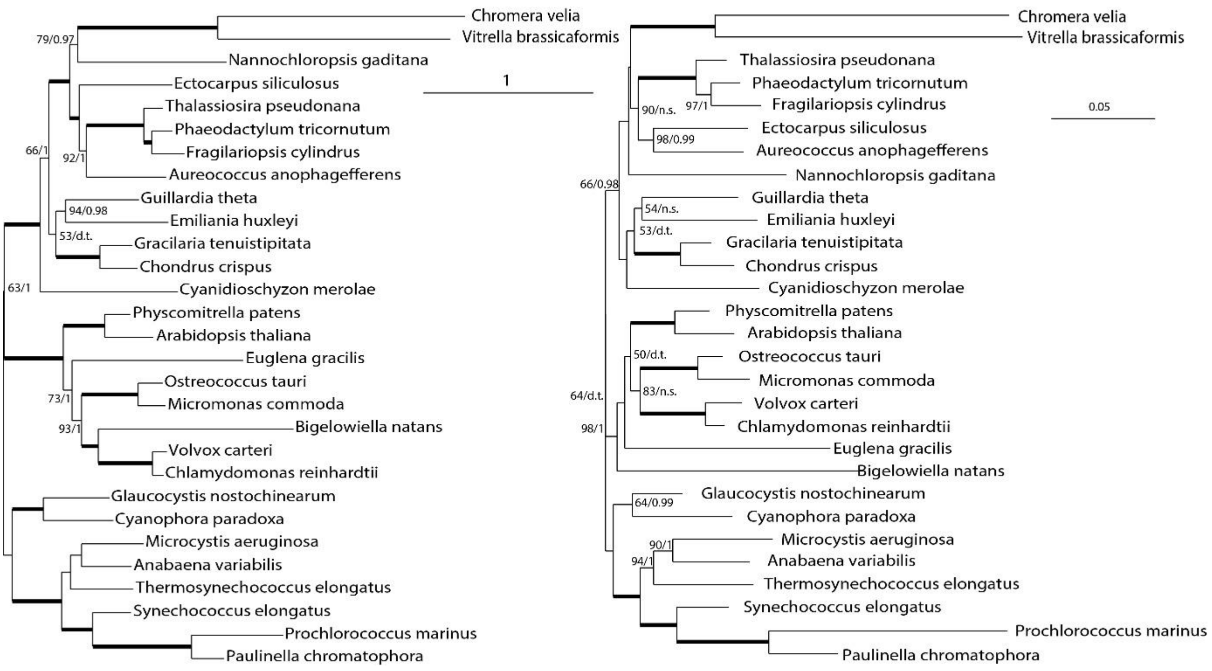

Supplementary Figure S1: Unrooted phylogenies derived from datasets of 55 thylakoid membrane complex proteins. A) Topology resulting from the analysis of a site-rich dataset of 10 465 sites. B) Topology resulting from the analysis of a site-poor dataset of 4872 sites. Support values are presented in the format ML bootstrap/Bayesian posterior probability. Branches with support values of 100/1.00 are shown in bold. The topologies shown here resulted from maximum likelihood analyses; branches where Bayesian analysis yielded different topology are indicated with "d.t." in the place of a Bayesian posterior probability; "n.s." indicates branches with low or non-existent support in the Bayesian analysis. Red plastid lineages and stramenopiles (inclusive of *Chromera velia* and *Vitrella brassicaformis*) were recovered in both analyses with moderate/high (ML bootstrap/Bayesian posterior probability) support. *Chromera* and *Vitrella* formed a sister group with the eustigmatophyte

Nannochloropsis gaditana with moderate support in the site-rich analysis, but this relationship was not recovered in the site-poor analysis.

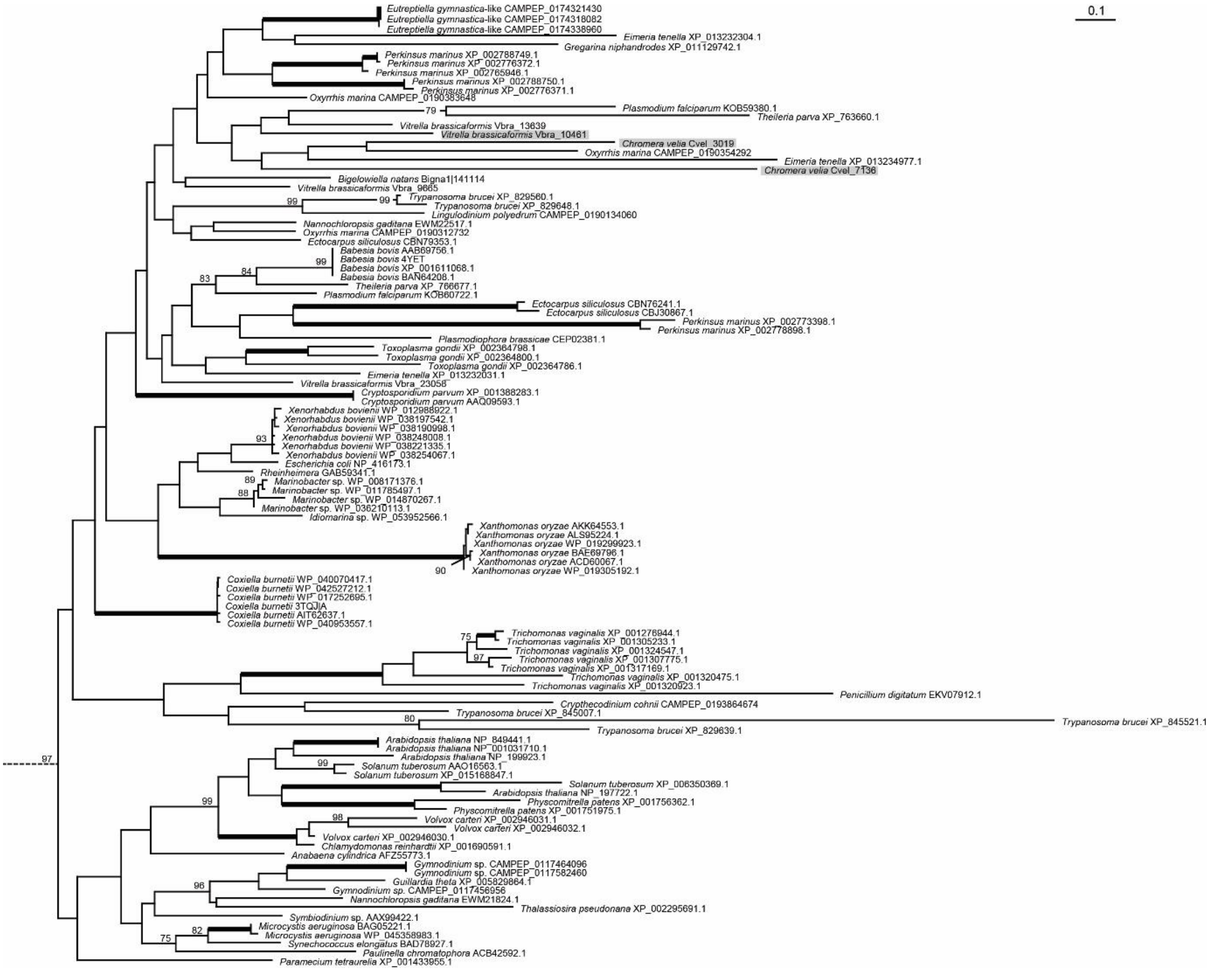

Supplementary Figure S2: Subtree from maximum likelihood phylogenetic analysis of manganese and iron superoxide dismutases (MnSODs and FeSODs, respectively). MnSODs and FeSODs resolved as two well-supported clades (ML bootstrap = 97); for clarity, only the clade formed by FeSODs is shown. Branches with ML support values of 100 are shown in bold; support values below 70 are not shown. PSI-associated FeSODs from *Chromera velia* are indicated by a circle; their phylogenetic positions are unsupported.

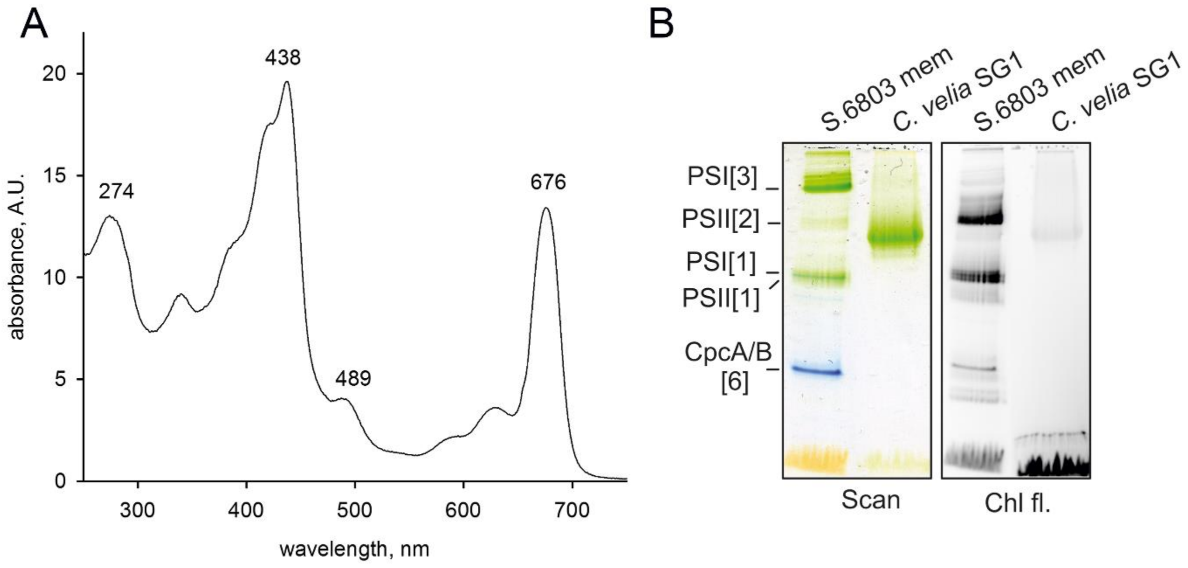

Supplementary Figure S3: Characterization of the purified *Chromera* PSI. A) Absorbance of the isolated PSI peak as recorded by a diode-array detector. B) The isolated PSI eluted separated by CN-PAGE. Solubilized membranes (3 µg of chlorophyll) from the cyanobacterium *Synechocystis* 6803 (*Syn* mem) were used to demonstrate the mobility of photosynthetic complexes. After separation, the clear native gel was photographed (scan) and scanned for chlorophyll fluorescence by LAS 4000 (Chl fl). PSI[1] (~350 kDa) and PSI[3] (~1 MDa), monomer and trimer of PSI, respectively; PSII[2] (~700 kDa), dimer of PSII; CpcA/B[6], ~100-kDa heterohexamer of CpcA and CpcB phycobiliproteins.

References

Curtis BA, Tanifuji G, Burki F, et al. 2012. Algal genomes reveal evolutionary mosaicism and the fate of nucleomorphs. Nature 492:59-65.

Edgar RC. 2004. MUSCLE: multiple sequence alignment with high accuracy and high throughput. Nucleic Acids Res. 32:1792-1797.

Emanuelsson O, Nielsen H, Brunak S, von Heijne G. 2000. Predicting subcellular localization of proteins based on their N-terminal amino acid sequences. J Mol Biol. 300:1005-1016.

Grigoriev IV, Nordberg H, Shabalov I, Aerts A, Cantor M, Goodstein D, Kuo A, Minovitsky S, Nikitin R, Ohm RA et al. 2012. The Genome Portal of the Department of Energy Joint Genome Institute. Nucleic Acids Res. 2012 Jan; 40 (Database issue): D26-32.

Gschloessl B, Guermeur Y, Cock JM. 2008. HECTAR: A method to predict subcellular targeting in heterokonts. BMC Bioinformatics 9:393.

Kanehisa M, Sato Y, Kawashima M, Furumichi M, Tanabe M. 2015. KEGG as a reference resource for gene and protein annotation. Nucleic Acids Res. 2015:1.

Larsson A. 2014. AliView: a fast and lightweight alignment viewer and editor for large data sets. Bioinformatics 30: 3276-3278.

Matsuzaki M, Misumi O, Shin-I T, Maruyama S, Takahara M, Miyagishima S, Mori T, Nishida K, Yagisawa F, Nishida K, et al. 2004. Genome sequence of the ultrasmall unicellular red alga *Cyanidioschyzon merolae* 10D. Nature 428:653-7.

Nielsen H, Engelbrecht J, Brunak S, von Heijne G. 1997. Identification of prokaryotic and eukaryotic signal peptides and prediction of their cleavage sites. Protein Eng. 10:1-6.

Nordberg H, Cantor M, Dusheyko S, Hua S, Poliakov A, Shabalov I, Smirnova T, Grigoriev IV, Dubchak I. 2014. The genome portal of the Department of Energy Joint Genome Institute: 2014 updates. Nucleic Acids Res. 42:D26-31.

Nowack ECM, Vogel H, Groth M, Grossman AR, Melkonian M, Glöckner G. 2011. Endosymbiotic gene transfer and transcriptional regulation of transferred genes in *Paulinella chromatophora*. Mol Biol Evol. 28:407-22.

Petersen TN, Brunak S, von Heihne G, Nielsen H. 2011. SignalP 4.0: discriminating signal peptides from transmembrane regions. Nat Methods. 8:785-786.

Stamatakis A, Hoover P, Rougemont J. 2008. A rapid bootstrap algorithm for the RAxML web-servers. Syst Biol. 75:758-771.

Woo YH, Ansari H, Otto TD, Klinger CM, Kolisko M, Michálek J, Saxena A, Shanmugam D, Tayyrov A, Veluchamy A, et al. 2015. Chromerid genomes reveal the evolutionary path from photosynthetic algae to obligate intracellular parasites. Elife 4:e06974.
